# Novel Cyclic di-GMP Effectors of the YajQ Protein Family Control Bacterial Virulence

**DOI:** 10.1371/journal.ppat.1004429

**Published:** 2014-10-16

**Authors:** Shi-qi An, Delphine L. Caly, Yvonne McCarthy, Sarah L. Murdoch, Joseph Ward, Melanie Febrer, J. Maxwell Dow, Robert P. Ryan

**Affiliations:** 1 Division of Molecular Microbiology, College of Life Sciences, University of Dundee, Dundee, United Kingdom; 2 School of Microbiology, Biosciences Institute, University College Cork, Cork, Ireland; 3 Genomic Sequencing Unit, College of Life Sciences, University of Dundee, Dundee, United Kingdom; Ohio State University, United States of America

## Abstract

Bis-(3′,5′) cyclic di-guanylate (cyclic di-GMP) is a key bacterial second messenger that is implicated in the regulation of many critical processes that include motility, biofilm formation and virulence. Cyclic di-GMP influences diverse functions through interaction with a range of effectors. Our knowledge of these effectors and their different regulatory actions is far from complete, however. Here we have used an affinity pull-down assay using cyclic di-GMP-coupled magnetic beads to identify cyclic di-GMP binding proteins in the plant pathogen *Xanthomonas campestris* pv. *campestris* (*Xcc*). This analysis identified XC_3703, a protein of the YajQ family, as a potential cyclic di-GMP receptor. Isothermal titration calorimetry showed that the purified XC_3703 protein bound cyclic di-GMP with a high affinity (K_d_∼2 µM). Mutation of *XC_3703* led to reduced virulence of *Xcc* to plants and alteration in biofilm formation. Yeast two-hybrid and far-western analyses showed that XC_3703 was able to interact with XC_2801, a transcription factor of the LysR family. Mutation of *XC_2801* and *XC_3703* had partially overlapping effects on the transcriptome of *Xcc*, and both affected virulence. Electromobility shift assays showed that XC_3703 positively affected the binding of XC_2801 to the promoters of target virulence genes, an effect that was reversed by cyclic di-GMP. Genetic and functional analysis of YajQ family members from the human pathogens *Pseudomonas aeruginosa* and *Stenotrophomonas maltophilia* showed that they also specifically bound cyclic di-GMP and contributed to virulence in model systems. The findings thus identify a new class of cyclic di-GMP effector that regulates bacterial virulence.

## Author Summary

Cyclic di-GMP is a bacterial second messenger that acts to regulate a wide range of functions including those that contribute to the virulence of pathogens. Our knowledge of the different actions and receptors for this nucleotide is far from complete. An understanding of the action of these elements may be key to interference with the processes they control. Here we have used an affinity pull-down assay using cyclic di-GMP-coupled magnetic beads to identify cyclic di-GMP binding proteins in the plant pathogen *Xanthomonas campestris*. This analysis identified XC_3703, a protein of the YajQ family that was able to bind cyclic di-GMP with high affinity. Mutation of *XC_3703* led to reduced virulence of *X. campestris* to plants and alteration in biofilm formation. Genetic and functional analysis of YajQ family members from the human pathogens *Pseudomonas aeruginosa* and *Stenotrophomonas maltophilia* showed that they also specifically bound cyclic di-GMP and contributed to virulence in model systems. The findings thus identify a new class of cyclic di-GMP effector that regulates bacterial virulence and raise the possibility that other members of the YajQ family, which occur widely in bacteria, also act in cyclic di-GMP signalling pathways.

## Introduction

Cyclic di-GMP (bis-(3′-5′) cyclic di-guanylate) is a second messenger in bacteria that acts to regulate a wide range of functions that include adhesion, biofilm formation, motility, synthesis of polysaccharides and synthesis of virulence factors in pathogens (recently reviewed by [1], [2], [3], [4]). The cellular level of cyclic di-GMP results from a balance between synthesis and degradation. Three protein domains are implicated in these processes: the GGDEF domain catalyzes synthesis of cyclic di-GMP from 2 molecules of GTP whereas EAL and HD-GYP domains catalyze hydrolysis of cyclic di-GMP, firstly to the linear nucleotide pGpG and then at different rates to GMP [1], [2], [3], [4]. All of these domains are named after conserved amino acid motifs. Most proteins with GGDEF/EAL/HD-GYP domains contain additional signal input domains, suggesting that their activities are responsive to signals or cues from the bacterial cell or its environment.

A number of cellular effectors or receptors for cyclic di-GMP have already been described in different bacteria [5], [6]. These include proteins with a PilZ domain, enzymatically inactive variants of GGDEF and EAL domains and a number of transcriptional regulators that do not possess a common domain organization [3], [5]. Furthermore, cyclic di-GMP is able to bind to untranslated regions of different mRNAs thereby affecting gene expression via riboswitches [3], [5]. Nevertheless, despite considerable progress, the mechanisms by which cyclic di-GMP exerts its action on diverse cellular processes remain incompletely understood, so that the discovery of further classes of cyclic di-GMP effectors is to be expected.

Here we have used an affinity pull down assay in order to identify potential cyclic di-GMP effectors in the phytopathogen *Xanthomonas campestris* pv. *campestris* (hereafter *Xcc*), the causal agent of black rot disease of cruciferous plants. As well as being a plant pathogen of global importance, *Xcc* is a model organism for molecular studies of plant-microbe interactions [7], [8]. *Xcc* has 37 proteins implicated in cyclic di-GMP synthesis and degradation and several of these are known to modulate synthesis of different virulence factors and virulence to plants [9], [10]. Thus far the only cyclic di-GMP effectors identified in *Xanthomonas* species are the transcription factor Clp, the enzymatically inactive GGDEF-EAL domain protein FimX and several PilZ domain proteins [5], [11], [12], [13], [14].

Our approach to reveal cyclic di-GMP binding proteins in *Xcc* described here identified XC_3703, a member of the YajQ family of proteins that is broadly distributed in bacteria. Mutational analysis showed that XC_3703 contributed to *Xcc* virulence to plants. Other members of the YajQ family from the human pathogens *Stenotrophomonas maltophilia* and *Pseudomonas aeruginosa* were also shown to preferentially bind cyclic di-GMP and to contribute to virulence in model systems. The findings thus identify a sub-group of the YajQ family of proteins as a new class of cyclic di-GMP effector.

## Results

### Identification of the cyclic di-GMP receptor protein XC_3703 in *Xcc*

**Figure 1:**
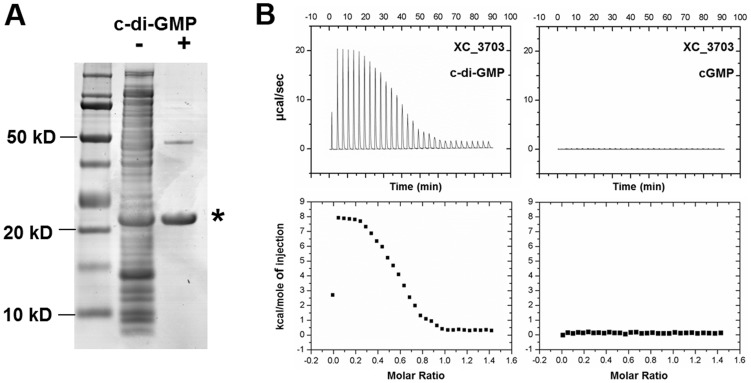
Identification of *Xanthomonas campestris* XC_3703 as a cyclic di-GMP- binding protein. (A) SDS-Polyacrylamide gel separation of *Xcc* proteins retained on cyclic di-GMP-coupled beads. Lanes from left: Molecular weight markers; Total crude extract of *Xcc* soluble proteins; Proteins retained by the cyclic di-GMP-coupled beads. The protein band indicated by the asterisk was identified by mass spectrometry of tryptic peptides as XC_3703. (B) Isothermal titration calorimetry analysis of binding of cyclic di-GMP and cyclic GMP by XC_3703. Lower panels show the integrated data obtained from the raw data, after subtracting the heat of dilution. Injection of cyclic di-GMP yielded an endothermic binding isotherm. Experimental data were fitted using the MicroCal ORIGIN version 7.0 software and a *K*_d_ for cyclic di-GMP was calculated as 2 µM. No binding of cyclic GMP to XC_3703 was detected.

To identify cyclic di-GMP receptor proteins, we performed an affinity pull-down assay using cyclic di-GMP–coupled magnetic beads and soluble protein extracts derived from the *Xcc* wild-type strain 8004. The selectively bound proteins were separated by SDS-polyacrylamide gel electrophoresis (Figure 1A) and were identified by peptide mass fingerprinting. Overall, 7 putative cyclic di-GMP binding proteins were identified from three cyclic di-GMP pull down experiments on *Xcc* 8004 lysates (Table S1). Three of these proteins were previously characterized cyclic di-GMP binding proteins from *Xcc*: the two PilZ domain-containing proteins XC_0965 and XC_3221 and the transcriptional regulator Clp, which is XC_0486. In addition, the analysis identified XC_1036, a protein containing a GGDEF domain with a predicted I-site (allosteric) cyclic di-GMP binding motif. Of particular interest to the work here was XC_3703 (MASCOT score 1156) a member of the highly conserved YajQ family of bacterial proteins. XC_3703 is related to YajQ of *Escherichia coli* (BLASTP *E* value is 10^−20^), a protein of unknown function that has motifs characteristic of nucleotide-binding proteins (Figure S1). YajQ family proteins are encoded by many bacterial genomes, to include both Gram-negative and Gram-positive bacteria. An amino acid sequence alignment of XC_3703 with sequences from a selected range of organisms is shown in Figure S1.

### XC_3703 binds cyclic di-GMP with high affinity

The ability of XC_3703 to bind different nucleotides including cyclic di-GMP was further examined using isothermal titration calorimetry (ITC) with the purified protein (*see* *Materials and Methods*). The full-length XC_3703 protein bound cyclic di-GMP with high affinity with a *K*_d_ of 2 µM (Figure 1B; Table S2). It appeared that binding was endothermic in nature, which is a characteristic of a binding mechanism that favours hydrogen bonding and hydrophobic interactions [15], [16]. The fitting of the binding isotherm data suggested a stoichiometry in which one molecule of cyclic di-GMP was bound by two molecules of the XC_3703 protein. The observed binding affinity is within the range of physiological cyclic di-GMP levels observed in *Xcc* and is similar to the binding affinity of a number of established cyclic di-GMP effector proteins. XC_3703 showed a much lower affinity for binding of ATP, GTP and cyclic di-AMP and no detectable binding of cyclic GMP or cyclic AMP (Figure 1B; Table S2). Taken together, these findings suggest that XC_3703 preferentially binds cyclic di-GMP.

### XC_3703 is required for virulence to plants and biofilm formation

**Figure 2:**
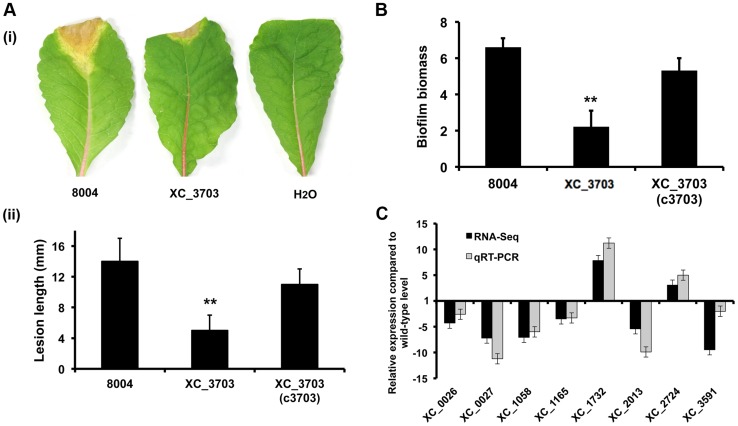
Transcriptome and phenotypic characterisation of an *XC_3703* deletion mutant reveals roles in biofilm formation and virulence in *Xcc*. (A) The virulence of the *Xcc* strains was tested by measurement of the lesion length after bacteria were introduced into the vascular system of Chinese Radish by leaf clipping. (i) Representative virulence assays for (from left to right) *Xcc* wild-type (8004), *XC_3703* deletion mutant and negative control (H_2_O). (ii) Re-integration of the gene encoding *XC_3703* into a neutral site in the mutant chromosome (indicated as *XC_3703* (cXC_3703)) restored the reduced virulence of the mutant towards wild-type. Values given are the mean and standard deviation of triplicate measurements each comprising of 30 leaves. Asterisks indicate values that are significantly different from the wild-type (p<0.01, two-tailed Student's *t*-test). (B) Deletion of *XC_3703* also led to decreased biofilm and cell adhesion on a glass surface when assessed by crystal violet staining. Biofilm biomass is measured as a ratio of absorbance at 550 and 600 nm. Re-integration of the gene encoding *XC_3703* into the chromosome (indicated as *XC_3703* (c3703)) restores the phenotypes towards wild-type. Values given are the mean and standard deviation of triplicate measurements. Asterisks indicate values that are significantly different from the wild-type (p<0.01, two-tailed Student's *t*-test). (C) Differential expression of selected genes implicated in virulence or biofilm formation in the *XC_3703* deletion mutant and wild-type as determined by RNA-Seq (dark grey) and qRT–PCR (light grey). Mutation of *XC_3703* affected transcript levels of *XC_0026* (cellulase), *XC_0027* (endoglucanase), *XC_1058* (pilin), *XC_1165* (TonB receptor) *XC_1732* (glycosyltransferase), *XC_2013* (MASE1 domain-containing protein), *XC_2724* (methyltransferase), and *XC_3591* (pectate lyase). The qRT–PCR data were normalised to 16S rRNA and is presented as the fold change with respect to the wild-type for each gene. Data (means ± standard deviation) are representative of four independent biological experiments. The complete RNA-Seq data set is detailed in Table S4.

Cyclic di-GMP is known to have a broad regulatory action in *Xcc* that includes influences on the synthesis of virulence factors and the formation of biofilms [7], [8]. The possible role of XC_3703 in these diverse regulatory actions was investigated by comparative phenotypic and transcriptomic analyses of the wild-type and *XC_3703* deletion mutant. The *XC_3703* mutant showed reduced virulence to Chinese Radish (Figure 2A) when tested by leaf clipping (*see* *Materials and Methods*) and reduced biofilm biomass when grown in complex medium (Figure 2B). Complementation restored these phenotypes towards wild-type.

Comparison of the transcriptome profiles of the wild-type and *XC_3703* mutant by RNA-Seq showed that deletion of *XC_3703* led to alteration in levels of transcript of a number of genes (Table S3, S4). These genes are associated with a range of biological functions that include bacterial motility and attachment, stress tolerance, virulence, regulation, transport, multidrug resistance, detoxification and signal transduction (Table S4).

The effect of *XC_3703* mutation on the level of transcript was validated by quantitative reverse transcription polymerase chain reaction (qRT-PCR) (Figure 2C). The genes selected for these analyses represented those that have been previously implicated in virulence and biofilm regulation in *Xcc* but with a range of fold change of expression and of diverse functional classes. The relative expression levels of 12 genes measured using qRT-PCR reflected the differences in gene expression observed by transcriptome analysis (Figure 2C).

### XC_3703 interacts with transcription regulator XC_2801

A number of cyclic di-GMP effector proteins exert their action through protein-protein interactions [6], [17]. To identify potential interacting proteins for XC_3703, we employed yeast two-hybrid (Y2H) analysis using the full-length XC_3703 protein as bait. Of the 36 preys isolated, the identities of 15 could be established by sequencing (Table S5). These represented partial or full-length proteins of diverse function including a putative octaprenyl-diphosphate synthase (XC_1377), a putative transcriptional regulator of the LysR family (XC_2801) and outer-membrane lipoprotein carrier protein (XC_2211). These particular protein interactions were confirmed when DNA fragments encoding the full length proteins were cloned and constructs were used individually as prey in Y2H analysis. The Y2H analysis was extended by Far-Western blotting experiments in which lysates of bacteria overexpressing the XC_3703 protein were separated by SDS polyacrylamide gel electrophoresis, transferred to nitrocellulose membranes and probed with the His6 tagged XC_1377, XC_2211 or XC_2801 which was then detected with an anti-His6 antiserum (Figure S2).

**Figure 3:**
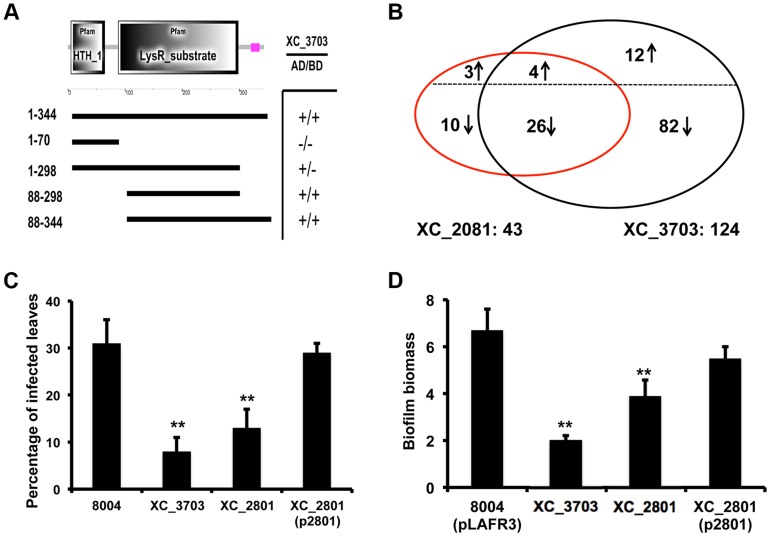
Protein interaction, transcription and phenotypic profiling reveal an overlap in regulatory influence of XC_3703 and XC_2801. (A) Domains of XC_2801 were predicted by SMART with amino acid positions indicated. Domain abbreviations are; HTH_1: helix-turn-helix DNA binding domain; LysR_substrate: small molecule binding domain associated with LysR family transcriptional regulators. Black lines below figures represent constructs cloned into either pBT or pTRG vectors to assess potential protein-protein interactions with XC_3703 using the bacterial two-hybrid assay. (+) indicates that an interaction was detected. (B) Venn diagrams showing the overlap of genes whose expression is down-regulated or up-regulated in the *XC_2801* or *XC_3703* mutant strain. Changes in gene expression in *XC_2801* and *XC_3703* deletion mutants compared with the wild-type *Xcc* were measured by RNA-Seq. Further detail of data analysis is given in Materials and Methods section and the complete RNA Seq data sets are detailed in Table S4. (C) Virulence of different *Xcc* strains assessed after spray inoculation. The percentage of the total number of inoculated leaves that showed typical black rot disease symptoms at the leaf margin is given. Values are means and standard deviations of three replicates, each comprising 25 plants and approximately 100 leaves. Asterisks indicate values that are significantly different from the wild-type at *p*<0.01 by two-tailed Student's *t*-test. Complementation of the *XC_2801* mutant by introduction of *XC_2801* cloned into pLAFR3 (indicated as XC_2801(p2801)) restored the phenotype towards wild-type. (D) Biofilm and cell adhesion of the *XC_2801* mutant to a glass surface was decreased compared to wild-type strain 8004. Complementation of the *XC_2801* mutant by introduction of *XC_2801* cloned into pLAFR3 (indicated as XC_2801(p2801)) restored the phenotype towards wild-type. Biofilm biomass was measured by crystal violet staining and expressed as a ratio of absorbance at 550/600 nm. Values given are the mean and standard deviation of triplicate measurements. Asterisks indicate values that are significantly different from the wild-type at *p*<0.01 by two-tailed Student's *t*-test.

Since XC_2801 gave the strongest interaction signal we continued analysis with this transcriptional regulator. XC_2801 comprises a conserved N-terminal DNA binding domain and C-terminal LysR substrate binding domain (Figure 3A). Bacterial two-hybrid analysis using different truncated versions of XC_2801 showed that only full-length XC_2801 and the derivatives retaining the substrate binding domain interacted with XC_3703, while no interaction was seen with the isolated DNA binding domain (Figure 3A).

### The regulatory action of XC_3703 overlaps with that of XC_2801

The findings outlined above indicate a potential interaction between XC_3703 and XC_2801. As a first step towards establishing a functional link between the two proteins, the phenotypic and transcriptional effects of mutation of *XC_2801* were compared with mutation of *XC_3703*.

Mutation of *XC_2801* significantly influenced the expression of 43 genes in *Xcc*; the regulated genes are associated with diverse functions that include extracellular enzyme production, attachment, biofilm formation and flagellar biosynthesis (Table S4). Quantitative RT-PCR methods were used to confirm alterations in expression of selected genes as revealed by RNA-Seq (Figure S3). Notably, comparison of the effects of mutation of *XC_3703* and *XC_2801* on the transcriptome revealed a significant overlap of regulatory influence (Figure 3B).

As outlined above, the *XC_3703* mutant showed reduced virulence in Chinese Radish when plants are inoculated by leaf clipping. In contrast, the *XC_2801* mutant showed no alteration in virulence when this method of inoculation was used. However, when plants were inoculated by spraying both *XC_3703* and *XC_2801* mutants showed reduced virulence, as indicated by a reduction in the percentage of inoculated leaves that develop black rot symptoms (Figure 3C). The effect of *XC_2801* mutation on virulence was less pronounced than that of *XC_3703* mutation however. Complementation of the *XC_2801* mutant by introduction of *XC_2801* cloned in pLAFR3 restored virulence to near wild-type level (Figure 3C).

Mutation of *XC_2801* also had an effect on *Xcc* biofilm formation, although the effect was not as pronounced as that following mutation of *XC_3703* (Figure 3D). Complementation with a clone expressing the full-length XC_2801 protein restored biofilm formation of the *XC_2801* mutant to near wild-type levels. Taken together, these observations support the contention the effects of XC_3703 are exerted, at least in part, by interaction with XC_2801.

### XC_2801 binds to target promoters only as a complex with XC_3703

**Figure 4:**
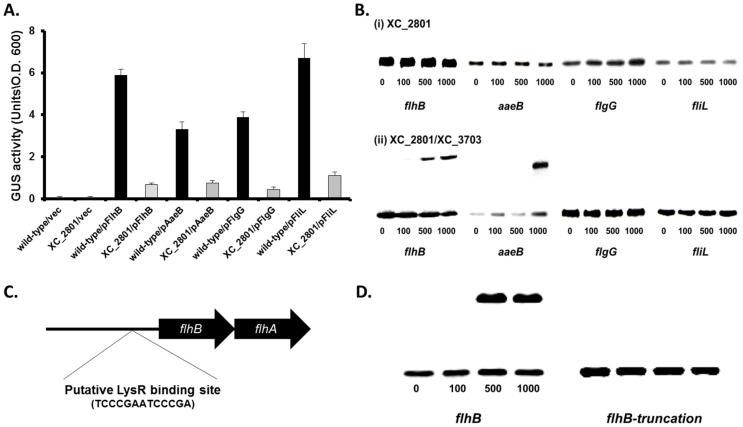
XC_2801 binds to specific promoters and only in the presence of XC_3703. (A) Promoter-fusion analysis of the upstream regions *flhB*, *aaeB*, *flgG* and *fliL* revealed significantly decrease in promoter activity in the *XC_2801* mutant strain. Values given are the mean and standard deviation of triplicate measurements. Values for promoter activity in the *XC_2801* mutant strain are significantly different from the wild-type at *p*<0.01 by two-tailed Student's *t*-test. The decrease in promoter activity was consistently observed in three independent experiments each consisting of three biological replicates. (B) Binding of XC_2801 to the promoter regions of *flhB*, *aaeB*, *flgG* and *fliL* in the absence (i) or presence (ii) of XC_3703 assessed by the use of electromobility shift assay (EMSA). Each lane contained 1.5 nM DIG-labelled Probe DNA, and in addition nanomolar concentrations of purified His6-tag XC_2801 protein as indicated below each well. (C) Bioinformatics reveals a putative LysR family regulator-binding site ( TCCCGAATCCCCGA ) located 80-67 nucleotides upstream of the predicted translational start site of *flhB*. (D) Although a shift was observed with the *flhB* upstream (promoter) region, no shift was observed with an overlapping DNA fragment that lacks the putative XC_2801 binding site (indicated as truncated *flhB*). The nanomolar concentration of His-tagged XC_2801 used in each assay is indicated below each well. DIG-labelled DNA at 1.5 nM was used.

The transcriptional analyses outlined above showed that levels of transcripts of the *flhB*, *aaeA*, *fliL* and *flgG* genes were decreased approximately fivefold in both the *XC_3703* and *XC_2801* mutant compared to the wild-type. The regulation of these genes by XC_2801 was also examined by the use of promoter fusions to *gusA* (*see* *Materials and Methods*). Differences in the level of GusA between the *XC_2801* mutant and wild-type were seen with all four fusions (Figure 4A), demonstrating that XC_2801 (directly or indirectly) regulates the expression of *flhB*, *aaeA*, *fliL* and *flgG*.

To further investigate the role of XC_2801 in regulation of transcription and any potential interplay between XC_2801 and XC_3703, electrophoretic mobility shift assays (EMSA) were used to examine protein binding to target promoters. His-tagged expression constructs of XC_2801 and XC_3703 were generated and both were subsequently purified by nickel affinity column chromatography. Purified His-tagged XC_2801 fusion protein caused no mobility shift of DNA probes spanning the upstream region of *flhB*, *aaeB*, *fliL* and *flgG* (Figure 4B). Interestingly, incubation of His-tagged XC_2801 together with XC_3703 led to a mobility shift of a DNA probe spanning the upstream regions of *flhB* and *aaeB* but not *fliC* or *flgG* (Figure 4B) whereas XC_3703 alone had no effect (Figure S4). These observations, taken together with the two-hybrid and far-western analyses presented above, suggest that under the conditions used, XC_2801 only binds to the upstream region of *flhB* and *aaeB* as a complex with XC_3703.

As LysR-type transcriptional regulators (LTTRs) typically bind a specific motif, T-N_11_-A, the upstream region of *flhBA* was examined for such a motif. The sequence TCCCGAATCCCGA was identified 80–67 bp upstream of the putative translational start site of *flhB* (Figure 4C). To investigate if this motif was essential for protein binding, EMSA was performed using an overlapping DNA fragment that lacked the full putative binding site. The presence of both XC_2801 and XC_3703 proteins did not cause a mobility shift of this DNA fragment demonstrating that XC_2801//XC_3703 binding requires the complete TCCCGAATCCCGA motif (Figure 4D).

### The binding of the XC_2801//XC_3703 complex to a target promoter is reversed by cyclic di-GMP

**Figure 5:**
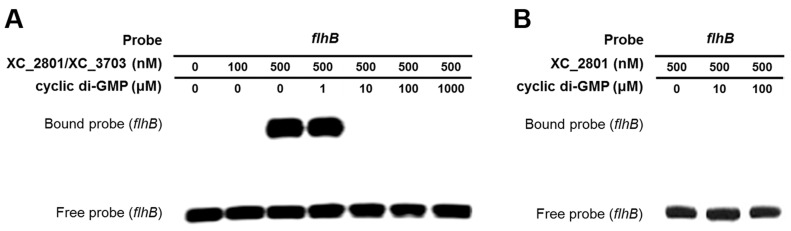
Binding of XC_2801//XC_3703 to the *flhBA* promoter is modulated by cyclic di-GMP. (A). The impact of various concentrations of cyclic di-GMP on the binding of XC_2801//XC_3703 to the *flhBA* promoter was assessed by the use of electromobility shift assay (EMSA). The DIG-labelled promoter fragment was incubated with purified XC_2801 and XC_3703 proteins and cyclic di-GMP. Concentrations of proteins and nucleotide are indicated above each well. (B) No binding of XC_2801 to the *flhBA* promoter region was seen in the presence of various concentrations of cyclic di-GMP as assessed by the use of EMSA. The DIG-labelled promoter fragment was incubated with purified XC_2801 and cyclic di-GMP. Concentrations of protein and nucleotide are indicated above each well.

As outlined above, XC_3703 preferentially binds cyclic di-GMP with a high affinity. This raised the question as to whether cyclic di-GMP could influence the binding of the XC_2801//XC_3703 complex to target promoters. To investigate this, the EMSA assay for the binding of XC_3703//XC_2801 to the *flhBA* promoter was repeated in the presence of added cyclic di-GMP at different concentrations. Addition of cyclic di-GMP at 1 µM had no apparent effect, but at 10 µM completely prevented DNA binding (Figure 5A). Other nucleotides did not have the same effect (Figure S4). XC_2801 alone did not bind to the *flhBA* promoter (as shown above) and addition of cyclic di-GMP did not alter this outcome (Figure 5B). Furthermore, XC_2801 appeared to have no affinity for the nucleotide as measured by ITC (Figure S5). These results suggest that cyclic di-GMP inhibits the interaction of the XC_3703//XC_2801 complex with DNA by binding to XC_3703, thus preventing the transcription of *flhBA*.

### XC_3703 homologues that bind cyclic di-GMP have roles in virulence in several human pathogens

Bioinformatic analysis reveals that proteins of the YajQ family are widely conserved in bacteria (Figure S1). To examine whether cyclic di-GMP binding was a common feature of this family, recombinant YajQ-like proteins from various bacteria including *Escherichia coli*, *Pseudomonas aeruginosa*, *Stenotrophomonas maltophilia*, *Bacillus cereus* and *Clostridium* species were purified and their ability to bind different nucleotides was assessed. Interestingly, affinity for cyclic di-GMP binding was only seen in YajQ family proteins from *P. aeruginosa* (PA4395) and *S. maltophilia* (Smlt4090) (Table S1). YajQ family proteins from *E. coli*, *Clostridium* sp. and *B. cereus* by contrast exhibited a greater affinity for ATP and/or GTP than for any cyclic mono- or di-nucleotide.

**Figure 6:**
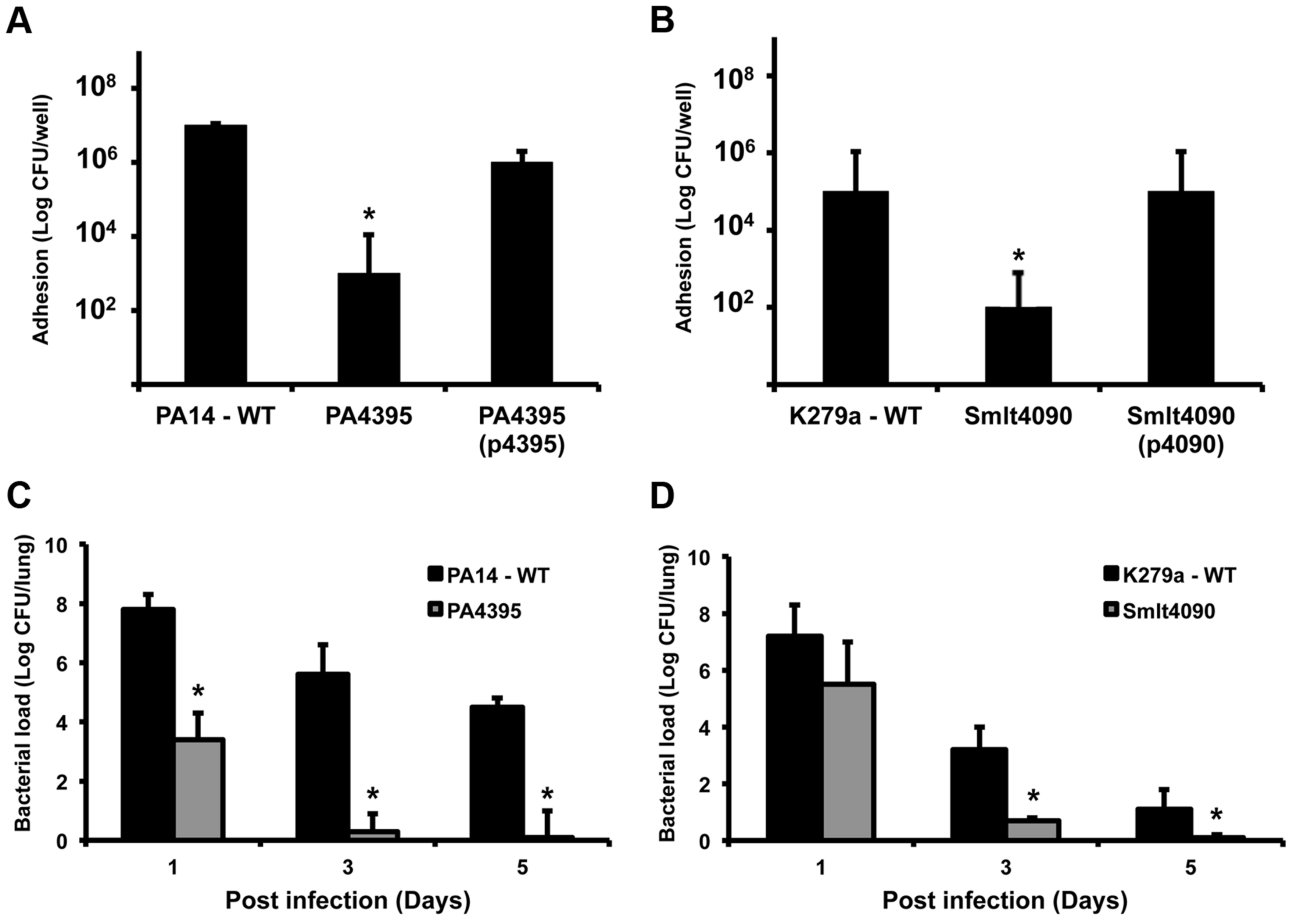
Roles for XC_3703 homologues in virulence of the human pathogens *Pseudomonas aeruginosa* and *Stenotrophomonas maltophilia*. Mutation of the genes encoding homologs of XC_3703 in *P. aeruginosa* (*PA4395*) and *S. maltophilia* (*Smlt4090*) resulted in strains with reduced capability to adhere to host cells and with reduced virulence in a murine model of infection. (A, B) Adhesion to human cells and internalization of strains of *P. aeruginosa* and *S. maltophilia*. Bronchial epithelial monolayers were co-cultured with ∼100 bacteria per cell for 2 hours. The bronchial epithelial cells were then washed and bacteria from cell lysates were plated on LB agar. Colonies were counted after 48 h. Values given are means and standard deviations of triplicate measurements. Asterisks indicate values that are significantly different from the appropriate wild-type (p<0.05, Student's t test). Complementation restored adhesion towards wild-type levels. (C, D). Mutation of *PA4395* in *P. aeruginosa* or *Smlt4090* in *S. maltophilia* leads to reduced colonization of mice compared to wild-type. C57BL/6 mice were intra-tracheally infected with 1×10^8^ CFU of wild-type *P. aeruginosa* PA14 or the *PA4395* mutant (C); wild-type *S. maltophilia* K279a or the *Smlt4090* mutant (D). Animals were sacrificed 1, 3 and 5 days after infection and 10-fold serial dilutions of lung homogenates were plated to determine the bacterial load. The means and standard deviations of triplicate measurements are shown. Asterisks indicate values that are significantly different from the appropriate wild-type (p<0.05, Student's t test).

The finding that XC_3703 can influence the virulence of *Xcc* prompted us to test whether PA4395 and Smlt4090 have a role in the virulence of *P. aeruginosa* and *S. maltophilia* respectively. Previous studies have established a correlation between the ability of strains of *P. aeruginosa* and *S. maltophilia* to attach and induce cytotoxicity in cultured mammalian cells and the virulence of these strains to mice [18], [19]. Mutation of either *PA4395* or *Smlt4090* led to reduced adhesion to monolayers of human bronchial epithelial cells compared to the wild-type (Figure 6 A, B). Importantly, the reduction in adhesion of these mutants could be restored to the level of the wild-type strain by complementation (Figure 6 A, B).

On the basis on these findings, we extended these studies to utilize the persistence mouse model of pulmonary infection. C57Bl/6 mice were intra-tracheally infected with wild-type *P. aeruginosa*, the *PA4395* mutant, wild-type *S. maltophilia* or the *Smlt4090* mutant and clearance of strains from the lung was examined at 1, 3 and 5 days post infection (*see* *Materials and Methods*). Both the *PA4395* and *Smlt4090* mutants had reduced persistence in C57BL/6 mice compared to their respective wild-type strains (Figure 6 C, D).

The findings from these infection models indicate that YajQ family proteins of *P. aeruginosa* and *S. maltophilia* significantly contribute to virulence of these opportunistic pathogens.

## Discussion

The YajQ family of proteins is broadly distributed in bacteria, with typically one member of this family in each species. YajQ family proteins have motifs characteristic of nucleotide or nucleic acid-binding proteins [16], [20]. Accordingly, YajQ from *Escherichia coli* has been shown to bind GTP and tRNA [16]. Here we show that some members of the YajQ family are novel cyclic di-GMP effectors that selectively bind this second messenger and act to regulate bacterial virulence. In particular we show that XC_3703 from *Xanthomonas campestris* influences the transcription of genes that contribute to virulence in plants and biofilm formation. We provide evidence that this action of XC_3703 is exerted, at least in part, through protein-protein interactions with the LysR family regulator XC_2801 in a manner that is negatively regulated by cyclic di-GMP. XC_3703 must exert a regulatory effect via additional pathways not involving XC_2801, but these currently remain unidentified.

We further identified the YajQ protein PA4395 of *P. aeruginosa* as a cyclic di-GMP binding protein. Previous studies to identify cyclic di-GMP-binding proteins from *P. aeruginosa* in a global fashion have used pull-downs with a cyclic di-GMP analog (2′-aminohexylcarbamoyl-cyclic di-GMP) covalently coupled to Sepharose beads [21] or a tri-functional capture compound incorporating cyclic di-GMP, biotin and a photo-activated reactive group [22]. Both of these studies identified a range of proteins, including six common proteins with an established function in cyclic di-GMP binding, but neither identified PA4395. Interestingly, of the novel proteins described in these two studies, only five were identified by both approaches, leading to the suggestion that the different compounds used for the experiments might be specific for a certain subset of proteins [22].

Knowledge of the cellular function of proteins of the YajQ family has been thus far restricted to the protein of *Pseudomonas syringae* that is implicated as a host factor controlling transcription in the bacteriophage Φ6 [23]. YajQ of *P. syringae* exerts an effect on phage RNA polymerase by protein-protein interactions involving the major protein (P1) of the phage core. Intriguingly this action is modulated in later phases of viral infection, even though YajQ is still present [23]. The underlying mechanism is unknown, although it has been suggested that a change in the chemical environment of the host cell between early and late phases of infection is responsible [23]. Our findings raise the possibility that one controlling factor could be the level of cyclic di-GMP in the bacterial cell.

In this study, XC_3703 was observed to bind cyclic di-GMP with an affinity of ∼2 µM. Measurements of cyclic di-GMP levels in wild-type *Xcc* strains under various conditions indicate that this value is within the physiological range of the concentration of the nucleotide [24], [25], [26]. The affinities of cyclic di-GMP effectors from a variety of organisms have been found to vary considerably, ranging from 10–15 µM down to ∼100 nM [27]. In *Xcc*, the transcription factor Clp binds cyclic di-GMP with an affinity of 3.5 µM, whereas the EAL domain of FimX has an affinity of 0.4 µM [11], [12]. The difference in binding affinities of these effectors has been suggested to represent a method whereby different responsive elements are selectively activated (or inactivated) as the cellular concentration of cyclic di-GMP changes [27].

Our experiments also show that not all members of the YajQ family preferentially bind cyclic di-GMP, suggesting that they have other cellular roles. This contention is supported by the observation that YajQ family members are found in bacteria that do not apparently have cyclic di-GMP signaling, such as *Haemophilus influenzae* [20], [28]. Proteins of the YajQ family show high level of amino acid sequence similarity but with some sequence divergence in the central part of the protein. Further work including structural studies will be required to identify residues that are critical for cyclic di-GMP binding, which may then allow bioinformatic discrimination of those family members that have this capability.

XC_3703 represents the first member of a new class of cyclic di-GMP binding protein and our findings suggest that the regulatory action of XC_3703 is exerted in part through protein-protein interaction with XC_2801, a protein of the LysR family that is the most abundant type of transcriptional regulator in bacteria [29]. Studies of members of the LysR family of transcriptional regulators indicate considerable structural flexibility, which may account for their variety of regulatory modes and versatility [30]. In general, the binding of small molecular weight molecules to the C-terminal co-inducer-binding domain modulates the activity of this family of regulators. XC_3703 also interacts with the C-terminal domain of XC_2801, suggesting a variation on the co-inducer mode of regulation (Figure S6). It appears that cyclic di-GMP specifically binds to XC_3703 with high affinity and that binding prevents the interaction of XC_3703//XC_2801 with a target promoter sequence (*flhBA*). In this manner, although XC_2801 alone does not bind cyclic di-GMP, transcriptional activation by XC_2801 is prevented when the concentration of cyclic di-GMP is high. Consistent with this view, the levels of expression of genes that we show here to be regulated by XC_2801 are reduced under conditions in which the cellular levels of cyclic di-GMP are high [30]. The absence of binding of XC_2801 or XC_2801//XC_3703 to the promoters of the *fliL* and *flgG* genes in EMSAs may indicate that the expression of these genes is indirectly regulated by XC_2801. We cannot however exclude that XC_2801 may also respond to a small-molecular weight co-inducer, which would be missing from these assays.

Notably, mutation of *XC_2801* affects expression of only a subset of the genes that are regulated by XC_3703. Furthermore, loss of XC_3703 influences virulence of *Xcc* when the bacteria are introduced into the leaf vascular system by leaf clipping, but loss of XC_2801 has no effect. From these observations we surmised that XC_3703 must exert additional regulatory effects, independently of XC_2801, that contribute to bacterial virulence. The Y2H analysis of proteins that interact with XC_3703 may give clues to the identity of such additional pathways. Intriguingly, interaction is seen between XC_3703 and HpaR1 (XC_2736), a GntR family transcriptional regulator that has been previously shown to control the hypersensitive response and virulence in *Xcc* [25]. Further work is required to determine the importance of this interaction to the virulence of *Xcc*.

Finally, we determined that YajQ proteins from the opportunistic human pathogens *P. aeruginosa* and *S. maltophilia* are capable of binding cyclic di-GMP and showed by mutational analysis that these proteins significantly influence virulence, biofilm formation and persistence in models of human infection. *S. maltophilia* is a xanthomonad, related to *Xcc* whereas *P. aeruginosa* is more distantly related. One challenge is to establish if regulatory interactions between proteins of the YajQ and LysR families have a role in cyclic di-GMP signaling in these other bacteria. Interference with cyclic di-GMP signaling has emerged as a promising approach towards treatment of bacterial biofilm formation and the control of virulence and disease. Potential targets include the enzymes involved in cyclic di-GMP synthesis or degradation and the effectors by which cyclic di-GMP exerts its action. Our knowledge of the mechanisms of cyclic di-GMP signaling and associated effectors is however incomplete, and further understanding will be key to the progress of a promising approach for disease control into effective therapeutic strategies.

## Materials and Methods

### Bacterial strains, plasmids, and culture conditions

*Xanthomonas campestris* pv *campestris* (*Xcc*) strains and culture conditions have been described previously [31], [32], [33]. Most experiments were carried out in NYGB medium, which comprises 5 g liter^−1^ bacteriological peptone (Oxoid, Basingstoke, U.K.), 3 g liter^−1^ yeast extract (Difco), and 20 g liter^−1^ glycerol. For biofilm formation, *Xcc* was grown in L medium, which comprises 10 g liter^−1^ bactotryptone (Difco), 5 g liter^−1^ yeast extract, 5 g liter^−1^ sodium chloride, and 1 g liter^−1^ D-glucose. *E. coli* strains were grown in LB medium at 37°C. Other plasmids and strains used are shown in Table S6. Where required antibiotics were used at concentrations of 100 µg ml^−1^ for ampicillin, 50 µg ml^−1^ for rifampicin, 20 µg ml^−1^ kanamycin and 15 µg ml^−1^ tetracycline.

### General molecular biology methods

Common molecular biological methods such as isolation of plasmid and chromosomal DNA, polymerase chain reaction (PCR), plasmid transformation as well as restriction digestion were carried out using standard protocols [34]. PCR products were cleaned using the Qiaquick PCR purification kit (Qiagen) and DNA fragments were recovered from agarose gels using Qiaquick minielute gel purification kit (Qiagen). Oligonucleotide primers were purchased from Sigma-Genosys.

### Construction of insertion and deletion mutant strains

In-frame deletion of selected genes was carried out using pK18*mobsac* as described previously for *XC_3703* and *XC_2801* [31], [32]. Mutants were also created by the disruption of genes with the use of the plasmid pK18*mob* as described previously for *Xcc* [31].

### Construction of protein expression vectors and protein purification

The DNA fragments encoding the proteins of interest were synthesized by Gene Oracle in pGOv4 and sub-cloned into pET47b or pLAFR3 before transformation into *E. coli* BL21 (DE3). Genomic regions are described in Table S7. BL21 (DE3) cells were grown in LB media and induced with 0.25 mM IPTG; protein overexpression was carried out at 37°C for 1 h. Purification was achieved by Ni^2+^ affinity chromatography using the N-terminal His6 tag followed by tag cleavage using recombinant 3C protease.

### Construction of *gusA* reporter plasmids

Reporter plasmids pG2277 (*flhB*), pG3487 (*aaeB*), pG2239 (*flgG*) and pG2262 (*fliL*) were constructed by cloning the putative promoter region and ribosome binding site (∼500-bp region upstream of the start codon) of *XC_2277*, *XC_3487*, *XC_2239* and *XC_2262* respectively into the broad-host-range cloning vector pLAFRJ, which harbors the coding region (without promoter and RBS) of β-glucuronidase (*gusA*) gene in its MCS (multiple cloning site). The putative promoter region and RBS of *XC_2277*, *XC_3487*, *XC_2239* and *XC_2262* were amplified from the chromosomal DNA of *Xcc*8004 using the primer pairs detailed in Table S7. The amplified DNA fragment, confirmed by sequencing, was inserted 9 bp upstream of the promoterless *gusA* ATG start codon in the vector pLAFRJ to create the recombinant plasmid. The recombinant plasmid obtained was further confirmed by restriction analysis and PCR. All reporter plasmids were introduced into the *Xcc* strains of interest through conjugation as previously described [25].

### Affinity pull-down assay

A volume of 50 ml of an *Xcc* culture with an OD_600_ of 1 was harvested and suspended in 1 ml 10 mM Tris·HCl (pH 7.5), 50 mM NaCl buffer containing EDTA-free complete protease inhibitor (Roche). Cells were mixed with 0.1-mm glass beads and lysed in a Fast-Prep machine twice for 45 seconds. Samples were centrifuged for 5 min at 17,000× *g* and subsequently for 1 h at 100,000× *g* to obtain cytoplasmic protein extracts. A volume of 50 ml streptavidin dynabeads (Invitrogen) coupled with 2.4 µM biotinylated cyclic-di-GMP (BioLog) were incubated with 1.2 mg cytoplasmic proteins in 1.5 mL 10% (vol/vol) glycerol, 1 mM MgCl_2_, 5 mM Tris (pH 7.5), 230 mM NaCl, 0.5 mM DTT, and 4 mM EDTA containing 50 µg/ml BSA for 30 min at room temperature. Samples were washed four times with the same buffer lacking BSA and suspended in 50 µl protein sample buffer. Samples were boiled for 5 min, beads removed, and 18 µl run on 12% (wt/vol) SDS/PAGE gels. Protein identification was carried out by in-gel proteolysis and the resultant peptides were analysed by LC-MS/MS on the LTQ Orbitrap Classic (FingersPrints, UK).

### Isothermal titration calorimetry (ITC) experiments

The dissociation constant (Kd) between wild-type XC_3703 (or selected homolog) with cyclic di-GMP, cyclic di-AMP, ATP, cyclic GMP or cyclic AMP was measured by using an VP-ITC MicroCalorimeter. Titrations were carried out at 25°C in an assay buffer containing 20 mM Tris-Cl (pH 8.0), 80 mM NaCl. Samples of XC_3703 for ITC measurements were dialyzed extensively against the assay buffer overnight. The concentrations of protein in the cell ranged between 10 to 300 µM and that of the nucleotide in the syringe was in the range of 1–4 mM. A volume of 8 µl of nucleotide was injected into the cell with a time lag of 180 s between each injection for a total of 30 times. ITC data were analyzed by integrating heat exchange amount after subtracting background dilution heat from the apparent values. Data fitting was based on a one-site binding model using the using the MicroCal ORIGIN version 7.0 software.

### RNA extraction and preparation for expression profiling

Three independent cultures of each selected *Xanthomonas* strain were sub-cultured and grown to logarithmic phase (0.7–0.8 OD_600_) at 30°C in NYGB broth without selection. 800 µl of RNA protect (Qiagen) was added to 400 µl culture and incubated at room temperature for 5 min. Cell suspensions were centrifuged, the supernatant was discarded, and pellets were stored at −80°C. After thawing, 100 µl TE-lysozyme (400 µg/ml) was added and samples were incubated at room temperature. Total RNA was isolated using the RNeasy Mini Kit (Qiagen) whereby cells were homogenised utilising a 20-gauge needle and syringe. Samples were treated with DNase (Ambion) according to manufacturer's instructions and the removal of DNA contamination was confirmed by PCR.

### Library construction and cDNA sequencing

RNA quality was assessed on a Bioanalyser PicoChip (Agilent) and RNA quantity was measured using the RNA assay on QuBit fluorometer (Life Technologies). Ribosomal RNA was depleted with Ribo-Zero rRNA Removal Kits for Gram-Negative Bacteria (Epicentre). The percentage of rRNA contamination was checked on a Bioanalyser PicoChip (Agilent). The rRNA-depleted sample was processed using the Illumina TruSeq RNA v2 sample preparation kit. In brief, the sample was chemically fragmented to ∼200 nt in length and the cleaved RNA fragments were primed with random hexamers into first strand cDNA using reverse transcriptase and random primers. The RNA template was removed and a replacement strand was synthesised to generate ds cDNA. The ds cDNA was end repaired to remove the overhangs from the fragmentation into blunt ends. A single ‘A’ nucleotide was added to the 3′ ends on the blunt fragments, which is complementary to a ‘T’ nucleotide on the 3′ end of the Illumina adapters. At this stage, adapters containing 6 nt barcodes were used for different samples to allow the pooling of multiple samples together. The resulted barcoded samples were enriched by 10 cycles of PCR to amplify the amount of DNA in the library. The final cDNA libraries were sequenced on an Illumina HiSeq2000 as per manufacturer's instructions. The RNA-Seq raw data files are accessible through XanthomonasGbrowse: http://browser.tgac.bbsrc.ac.uk/cgi-bin/gb2/gbrowse/Xanthomonas_8004/.

### Computational analysis

Cluster generation was performed using the Illumina cBot and the cDNA fragments were sequenced on the Illumina HiSeq2000 following a standard protocol. The fluorescent images were processed to sequences using the Real-Time Analysis (RTA) software v1.8 (Illumina). Raw sequence data were demultiplexed by assigning the sequenced reads of each sample with their corresponding indexed reads. The reads for each sample were then processed through the primary analysis pipeline to generate FASTQ files.

#### Read mapping, annotation and quantification of transcript levels

Reads for each sample were aligned to the *Xanthomonas campestris* pv *campestris* 8004 NC_007086 assembly (Integrated Microbial Genomes (IMG) database, taxon object ID 637000343) using Bowtie version 0.12.7 with default parameters. Transcript abundance was determined for the gene models annotated in the IMG *Xanthomonas campestris* pv *campestris* 8004 genome release IMG/W 2.0 using the Bowtie RNA-Seq BAM alignments for each of the samples. To estimate the level of transcription for each gene, the number of reads that mapped within each annotated coding sequence (CDS) was determined.

#### Analysis of differential expression

Differential expression was assessed using Cufflinks (Cufflinks reports an expression value for each transcript and gene in Fragments Per Kilobase of exon model per Million mapped fragments (FPKM). A test statistic is also calculated which, after Benjamini-Hochberg correction for multiple-testing was used to determine significant changes in expression between each pair of samples (false discovery rate 0.05).

### Quantitative Real-time PCR

Quantitative RT-PCRs were used to validate RNA-Seq data. Reverse transcription PCR was achieved using a cDNA synthesis kit (Promega) according to the manufacturer's instructions. Specific RT-PCR primers were used to amplify central fragments of approximately 200 bp in length from different genes. For qRT-PCRs, quantification of gene expression and melting curve analysis were completed using a LightCycler (Roche) and Platinum SYBR Green qPCR Supermix-UGD (Invitrogen) was used according to manufacturer's instructions. The constitutively expressed housing keeping gene, *16S rRNA* was used as a reference to standardize all samples and replicates.

### Yeast two-hybrid screen

The methods used for construction and analysis of the *Xcc* genomic DNA prey library in the plasmid vector pOAD were as described previously [17]. *Xcc* DNA sequence coding for the XC_3703 used as bait was amplified by PCR using *Xcc* genomic DNA as template and primers designed based on the *Xcc* genome sequence. These primers were designed to contain unique restriction sites to facilitate cloning into the pOBD vector downstream of the Gal4 DNA-binding domain. After transformation into *E. coli* DH5α cells, individual colonies were picked for plasmid isolation and confirmation by DNA sequencing. The bait construct was transformed into *Saccharomyces cerevisiae*, and this strain was used to screen the *Xcc* genomic DNA prey library. Approximately 2×10^8^ yeast transformants (carrying both and prey vectors) were plated on selective media that lacked adenine, histidine, tryptophan, and leucine. Fifteen hundred transformants displaying prototrophy were transferred to plates with adenine and histidine but lacking tryptophan and leucine. Preys were identified by isolation of plasmids and sequencing.

For measurement of interactions of the segments of XC_2801 with XC_3703, doubly transformed yeast strains were grown in YPD medium (10 g liter^−1^ Bacto-yeast extract (Difco), 20 g liter^−1^ Bactopeptone (Difco), and 2 g liter^−1^ D-glucose) were performed as described [17].

### Far-western assays

*In vitro* binding assays were performed as described previously [34]. Briefly, ≈20 µg of purified recombinant proteins or total lysates of *E. coli* cultures expressing recombinant proteins were resolved by SDS/PAGE (12% acrylamide) and transferred to nitro- cellulose membranes. The membranes were blocked with TBS-TTmilk [140 mM NaCl, 20 mM TrisHCl (pH 7.4), 0.1% Triton X-100, 0.1% Tween-20, 5% powdered milk] for 1 h and then probed with anti-His6 antiserum as described previously [35].

### Electrophoretic gel mobility shift assay

The DNA probes used for EMSA were prepared by PCR amplification of the desired upstream region of *flhB*, using oligonucleotides as the primers (primers and amplified regions are described in Table S7). The purified PCR products were 3′-end-labelled with digoxigenin following the manufacturer's instruction (Roche). The EMSA was carried out using the DIG Gel Shift Kit 2nd Generation (Roche) as recommended by the manufacturer with some modifications. Ten fmoles of the DIG-labelled fragment and a range of 0–1000 nM of XC_2801 protein were added to the binding reaction. The mixture was allowed to proceed at room temperature for 45 min. The samples were separated by electrophoresis on 6% native polyacrylamide gels and transferred to Hybond-N blotting membrane (Amersham). Protein–DNA complexes were visualized by NBT/BCIP according to the manufacturer's instructions (Roche).

### Plant virulence assays

The virulence of *Xcc* to Chinese Radish was estimated after bacteria were introduced into the leaves by leaf clipping and spraying as previously detailed [10], [31], [36]. For leaf clipping assay, bacteria grown overnight in NYGB medium were washed and re-suspended in water to an OD at 600 nm of 0.001. For leaf clipping the last completely expanded leaf was cut with scissors dipped in the bacterial suspensions. Thirty leaves were inoculated for each strain tested. Lesion length was measured 14 days after inoculation. Each strain was tested in at least four separate experiments. For spraying assays, five-week old seedlings with fully expanded leaves were used. Bacteria were grown overnight in NYGB medium, centrifuged and re-suspended in water to a cell density OD of 0.01 at 600 nm for spraying. A volume of 50 ml of the bacterial suspension was inoculated onto the leaves of 25 plants (approximately 100 leaves) by spraying. Three replicates of each independent experiment were carried out. Ten days after inoculation the relative virulence was determined as the percentage of the total inoculated leaves that showed the typical black rot disease symptoms at the leaf margin. The experiment was repeated three times.

### Biofilm assays

Biofilm was assessed by attachment to glass and was determined by crystal violet staining. Log-phase-grown bacteria were diluted to OD_600_ nm = 0.02 in L media broth and 5 ml was incubated at 30°C for 24 h in 14-ml glass tubes. After gently pouring off the media, bacterial pellicles were washed twice with water and were then stained with 0.1% crystal violet. Tubes were washed and rinsed with water until all unbound dye was removed [25], [31]. Bound crystal violet was eluted in ethanol and measured at OD_595_. Three independent assays were carried out for each strain.

### Ethics statement

Animal experiments were conducted in compliance with a protocol (# 16-051-11) approved by The Animal Experimentation Ethics Committee (AEEC) of University College Cork or Cork University Hospital. The Minister for Health accredits the University College Cork for conducting animal experiments. AEEC is guided by legislative requirements, in particular the Cruelty to Animals Act as amended and supplemented by the European Communities.

### Infection and treatment of animals

Mouse infection was performed using a variation on the mouse model described by [35], [37]. Briefly, *P. aeruginosa* or *S. maltophilia* strains were grown in Luria broth at 37°C overnight with shaking, after which bacteria were collected by centrifugation and resuspended in PBS. The exact number of bacteria was determined by plating serial dilutions of each inoculum on Luria broth agar plates. Female C57BL/6 mice (approximately 8-weeks old) were anesthetized and infected by the intratracheal route with 20 µl of culture of wild-type strains or mutants at a final inoculum of 5×10^6^ colony forming unit (CFU) per mouse. Mice were killed at 1, 3 and 5 days post-infection by intraperitoneal injection of 0.3 ml of 30% pentobarbital. Lungs and spleens were harvested aseptically and homogenized in sterile PBS. A 10-fold serial dilution of lung homogenates was plated on *Pseudomonas* isolation agar (for *P. aeruginosa* strains) or NGBA media (for *S. maltophilia* strains). The results (means ± standard deviation) are expressed as CFU/organ.

### Epithelial cell co-culture

*P. aeruginosa* or *S. maltophilia* strains were grown on bronchial cells using a co-culture model system as previously described [37], [38], [39]. Briefly, bronchial epithelial monolayers were co-cultured with 100 bacteria per cell for 2 hours. The bronchial epithelial cells were then washed and bacteria from cell lysates were plated on LB agar. Colonies were counted after 48 hours.

## Supporting Information

Figure S1 Sequence alignment of proteins from the YajQ family using CLC workbench software. Residues with similar properties are boxed within the same color. Key: *Xanthomonas campestris* (XC_3703), *Escherichia coli* (YajQ), *Bacillus cereus* (BCK_02545), *Bordetella pertussis* (BP1193_10170), *Bacillus subtilis* (YitK), *Clostridium cellulovorans* (Clocel_3875), *C. jejuni* (BN867_03480), *Enterococcus faecalis* (EFD32_0973), *Haemophilus influenzae* (R2846_1298), *Legionella pneumophila* (LPE509_01999), *Mycobacterium tuberculosis* (MT0592), *Pseudomonas aeruginosa* (PA4395), *Pseudomonas syringae* (PSPPH_4093), *Stenotrophomonas maltophilia* (Smlt4090), *Vibrio cholerae* (VC_1508), *Yersinia pestis* (YPC_3455). Sequence alignment of proteins from the YajQ family. Consensus residues are listed below the alignment. The sequence logo illustrates the conservation between sequences. Where the height of each letter reflects the relative frequency of the corresponding amino acid at that position; the overall height of the column reflects the degree of sequence conservation at that position. On the top of the sequences are indicated the secondary structure elements as Quick2D (http://toolkit.tuebingen.mpg.de/quick2_d) and the residues predicted to be involved in interaction with tRNA are labeled with “+”.
(TIF)

Figure S2 Far-Western analysis showing interactions of XC_3703 with XC_1377, XC_2211 and XC_2801. Lysates of *Xcc* overexpressing the XC_3703 protein were separated by SDS polyacrylamide gel electrophoresis, transferred to nitrocellulose membranes and separate blots were probed with His6 tagged XC_1377, XC_2211 or XC_2801 which was then detected with an anti-His6 antiserum. The left lane on each blot (H_2_O) represents a blank control with no His-tagged protein. Far–western signals were seen with XC_1377, XC_2801 and XC_2211.
(TIF)

Figure S3 Differential expression of selected genes implicated in virulence or biofilm formation in the *XC_2801* deletion mutant and wild-type as determined by RNA-Seq (dark grey) and qRT–PCR (light grey). Mutation of *XC_2801* affected transcript levels of *XC_0026* (cellulase), *XC_1292* (endoproteinase), *XC_1732* (glycosyltransferase), *XC_2277* (flagellar biosynthesis protein), *XC_2278* (flagellar biosynthesis protein), *XC_2458* (endomannosidase). The qRT–PCR data were normalised to 16S rRNA and is presented as the fold change with respect to the wild-type for each gene. Data (means ± standard deviation) are derived from four independent biological experiments. The complete RNA-Seq data set is detailed in Table S3; S4.
(TIF)

Figure S4 (A) Promoter regions of *flhB* and *aaeB* showed no mobility in presence of XC_3703 alone as assessed by electromobility shift assay (EMSA). Each lane contained 1.5 nM DIG-labelled Probe DNA, and in addition to 500 nM of purified His6-tag XC_3703 protein. (B) Binding of XC_2801//XC_3703 complex to the *flhBA* promoter in the presence of various nucleotides. EMSA assessment of the impact of the presence of nucleotides AMP, GTP, cyclic GMP, cyclic di-AMP, ATP or cyclic AMP at 10 µM on the binding of XC_2801//XC_3703 to the *flhBA* promoter. DIG-labelled promoter fragments were incubated with purified XC_2801//XC_3703 proteins in the absence or presence of nucleotide as indicated.
(TIF)

Figure S5 Assessment of binding of cyclic di-GMP and cyclic GMP by XC_2801 using isothermal titration calorimetry. Panels show the integrated data obtained from the raw data, after subtracting the heat of dilution. Experimental data were fitted using the MicroCal ORIGIN version 7.0 software. XC_2801 has no apparent affinity for cyclic di-GMP.
(TIF)

Figure S6 Model for the regulation of gene expression by XC_2801, XC_3703 and cyclic di-GMP. DNA binding and the transcriptional activity of XC_2801 is promoted by binding of XC_3703 to the LysR_substrate-binding domain. The binding of cyclic di-GMP to XC_3703 inhibits this protein-protein complex from interaction with DNA, thereby leading to a reduction in gene transcription. We cannot exclude however that the binding of cyclic di-GMP by XC_3703 prevents the interaction with XC_2801, which cannot bind the promoter alone. XC_3703 alone does not bind to the promoter DNA and XC_2801 does not bind cyclic di-GMP.
(TIF)

Table S1 Proteins that were identified in three cyclic di-GMP pull down experiments.
(DOCX)

Table S2 Binding constants of XC_3703 and other YajQ-like proteins for different nucleotides as determined by isothermal titration calorimetry.
(DOCX)

Table S3 Summary of sequencing data for the *Xcc* cDNA samples analysed.
(DOCX)

Table S4 List of genes expressed in *XC_2801* and *XC_3703* mutant backgrounds compared to wild-type. Significantly differentially expressed genes (fold change ≥3) were determined using Cufflinks after Benjamini-Hochberg correction. The fold change is the ratio of mutant fragments per kilobase of exon per million fragments mapped (FPKM) to wild-type FPKM.
(DOCX)

Table S5 Summary of protein–protein interactions involving XC_3703 observed in this study.
(DOCX)

Table S6 Strains and plasmids used in this study.
(DOCX)

Table S7 Primers used in this study.
(DOCX)

## References

[1] Boyd CD, O'Toole GA (2012) Second messenger regulation of biofilm formation: Breakthroughs in understanding c-di-GMP effector systems. Annu Rev Cell Dev Biol 28: 439–462. 23057745 10.1146/annurev-cellbio-101011-155705 PMC4936406

[2] Hengge R (2009) Principles of c-di-GMP signalling in bacteria. Nat Rev Microbiol 7: 263–273. 19287449 10.1038/nrmicro2109

[3] Romling U, Galperin MY, Gomelsky M (2013) Cyclic di-GMP: the first 25 years of a universal bacterial second messenger. Microbiol Mol Biol Rev 77: 1–52. 23471616 10.1128/MMBR.00043-12 PMC3591986

[4] Schirmer T, Jenal U (2009) Structural and mechanistic determinants of c-di-GMP signalling. Nat Rev Microbiol 7: 724–735. 19756011 10.1038/nrmicro2203

[5] Ryan RP, Tolker-Nielsen T, Dow JM (2012) When the PilZ don't work: effectors for cyclic di-GMP action in bacteria. Trends Microbiol 20: 235–242. 22444828 10.1016/j.tim.2012.02.008

[6] Sondermann H, Shikuma NJ, Yildiz FH (2012) You've come a long way: c-di-GMP signaling. Curr Opin Microbiol 15: 140–146. 22226607 10.1016/j.mib.2011.12.008 PMC3320698

[7] Mansfield J, Genin S, Magori S, Citovsky V, Sriariyanum M, Ronald P, Dow M, Verdier V, Beer SV, Machado MA, et al (2012) Top 10 plant pathogenic bacteria in molecular plant pathology. Mol Plant Pathol 13: 614–629. 22672649 10.1111/j.1364-3703.2012.00804.x PMC6638704

[8] Ryan RP (2013) Cyclic di-GMP signalling and the regulation of bacterial virulence. Microbiol-SGM 159: 1286–1297. 10.1099/mic.0.068189-0 PMC3749722 23704785

[9] Ryan RP, Fouhy Y, Lucey JF, Crossman LC, Spiro S, He YW, Zhang LH, Heeb S, Camara M, Williams P, et al (2006) Cell-cell signaling in *Xanthomonas campestris* involves an HD-GYP domain protein that functions in cyclic di-GMP turnover. Proc Natl Acad Sci USA 103: 6712–6717. 16611728 10.1073/pnas.0600345103 PMC1458946

[10] Ryan RP, Fouhy Y, Lucey JF, Jiang B-L, He Y-Q, Feng J-X, Tang J-L, Dow JM (2007) Cyclic di-GMP signalling in the virulence and environmental adaptation of *Xanthomonas campestris* . Mol Microbiol 63: 429–442. 17241199 10.1111/j.1365-2958.2006.05531.x

[11] Chin K-H, Lee Y-C, Tu Z-L, Chen C-H, Tseng Y-H, Yang J-M, Ryan RP, McCarthy Y, Dow JM, Wang AHJ, et al (2010) The cAMP receptor-like protein CLP is a novel c-di-GMP receptor linking cell-cell signaling to virulence gene expression in *Xanthomonas campestris* . J Mol Biol 396: 646–662. 20004667 10.1016/j.jmb.2009.11.076

[12] Guzzo CR, Dunger G, Salinas RK, Farah CS (2013) Structure of the PilZ-FimX(EAL)-c-di-GMP complex responsible for the regulation of bacterial type IV pilus biogenesis. J Mol Biol 425: 2174–2197. 23507310 10.1016/j.jmb.2013.03.021

[13] McCarthy Y, Ryan RP, O'Donovan K, He Y-Q, Jiang B-L, Feng J-X, Tang J-L, Dow JM (2008) The role of PilZ domain proteins in the virulence of *Xanthomonas campestris* pv. *campestris* . Mol Plant Pathol 9: 819–824. 19019010 10.1111/j.1364-3703.2008.00495.x PMC6640328

[14] Tao F, He Y-W, Wu D-H, Swarup S, Zhang L-H (2010) The cyclic nucleotide monophosphate domain of *Xanthomonas campestris* global regulator Clp defines a new class of cyclic-di-GMP effectors. J Bacteriol 192: 1020–1029. 20008070 10.1128/JB.01253-09 PMC2812978

[15] Lundback T, Hard T (1996) Sequence-specific DNA-binding dominated by dehydration. Proc Natl Acad Sci USA 93: 4754–4759. 8643475 10.1073/pnas.93.10.4754 PMC39351

[16] Saveanu C, Miron S, Borza T, Craescu CT, Labesse G, Gagyi C, Popescu A, Schaeffer F, Namane A, Laurent-Winter C, et al (2002) Structural and nucleotide-binding properties of YajQ and YnaF, two *Escherichia coli* proteins of unknown function. Protein Sci 11: 2551–2560. 12381839 10.1110/ps.0217502 PMC2373726

[17] Ryan RP, McCarthy Y, Kiely PA, O'Connor R, Farah CS, Armitage JP, Dow JM (2012) Dynamic complex formation between HD-GYP, GGDEF and PilZ domain proteins regulates motility in *Xanthomonas campestris* . Mol Microbiol 86: 557–567. 22924852 10.1111/mmi.12000

[18] Fouhy Y, Scanlon K, Schouest K, Spillane C, Crossman L, Avison MB, Ryan RP, Dow JM (2007) Diffusible signal factor-dependent cell-cell signaling and virulence in the nosocomial pathogen *Stenotrophomonas maltophilia* . J Bacteriol 189: 4964–4968. 17468254 10.1128/JB.00310-07 PMC1913462

[19] Mulcahy H, O'Callaghan J, O'Grady EP, Adams C, O'Gara F (2006) The posttranscriptional regulator RsmA plays a role in the interaction between Pseudomonas aeruginosa and human airway epithelial cells by positively regulating the type III secretion system. Infect Immun 74: 3012–3015. 16622241 10.1128/IAI.74.5.3012-3015.2006 PMC1459696

[20] Teplyakov A, Obmolova G, Bir N, Reddy P, Howard AJ, Gilliland GL (2003) Crystal structure of the YajQ protein from *Haemophilus influenzae* reveals a tandem of RNP-like domains. J Struct Funct Genomics 4: 1–9. 12943362 10.1023/a:1024620416876

[21] Duevel J, Bertinetti D, Moeller S, Schwede F, Morr M, et al (2012) A chemical proteomics approach to identify c-di-GMP binding proteins in *Pseudomonas aeruginosa* . J Microbiol Methods 88: 229–236. 22178430 10.1016/j.mimet.2011.11.015

[22] Nesper J, Reinders A, Glatter T, Schmidt A, Jenal U (2012) A novel capture compound for the identification and analysis of cyclic di-GMP binding proteins. J Proteomics 75: 4874–4878. 22652488 10.1016/j.jprot.2012.05.033

[23] Qiao X, Sun Y, Qiao J, Mindich L (2008) The role of host protein YajQ in the temporal control of transcription in bacteriophage Phi 6. Proc Natl Acad Sci USA 105: 15956–15960. 18836083 10.1073/pnas.0807489105 PMC2572959

[24] Ha D-G, Merritt JH, Hampton TH, Hodgkinson JT, Janecek M, Spring DR, Welch M, O'Toole GA (2011) 2-Heptyl-4-Quinolone, a precursor of the *Pseudomonas* quinolone signal molecule, modulates swarming motility in *Pseudomonas aeruginosa* . J Bacteriol 193: 6770–6780. 21965567 10.1128/JB.05929-11 PMC3232867

[25] Lu X-H, An S-Q, Tang D-J, McCarthy Y, Tang J-L, Dow JM, Ryan RP (2012) RsmA regulates biofilm formation in *Xanthomonas campestris* through a regulatory network involving cyclic di-GMP and the Clp transcription factor. PLoS One 7: 12. 10.1371/journal.pone.0052646 PMC3528676 23285129

[26] Ryan RP, Lucey J, O'Donovan K, McCarthy Y, Yang L, Tolker-Nielsen T, Dow JM (2009) HD-GYP domain proteins regulate biofilm formation and virulence in *Pseudomonas aeruginosa* . Environ Microbiol 11: 1126–1136. 19170727 10.1111/j.1462-2920.2008.01842.x

[27] Pultz IS, Christen M, Kulasekara HD, Kennard A, Kulasekara B, Miller SI (2012) The response threshold of *Salmonella* PilZ domain proteins is determined by their binding affinities for c-di-GMP. Mol Microbiol 86: 1424–1440. 23163901 10.1111/mmi.12066 PMC5034864

[28] Galperin MY (2010) Diversity of structure and function of response regulator output domains. Curr Opin Microbiol 13: 150–159. 20226724 10.1016/j.mib.2010.01.005 PMC3086695

[29] Maddocks SE, Oyston PCF (2008) Structure and function of the LysR-type transcriptional regulator (LTTR) family proteins. Microbiol-SGM 154: 3609–3623. 10.1099/mic.0.2008/022772-0 19047729

[30] Momany C, Neidle EL (2012) Defying stereotypes: the elusive search for a universal model of LysR-type regulation. Mol Microbiol 83: 453–456. 22235937 10.1111/j.1365-2958.2011.07960.x

[31] An S-Q, Febrer M, McCarthy Y, Tang D-J, Clissold L, Kaithakottil G, Swarbreck D, Tang J-L, Rogers J, Dow JM, et al (2013) High-resolution transcriptional analysis of the regulatory influence of cell-to-cell signalling reveals novel genes that contribute to *Xanthomonas* phytopathogenesis. Mol Microbiol 88: 1058–1069. 23617851 10.1111/mmi.12229 PMC3744752

[32] An S-Q, Lu G-T, Su H-Z, Li R-F, He Y-Q, Jiang B-L, Tang D-J, Tang J-L (2011) Systematic mutagenesis of all predicted *gntR* genes in *Xanthomonas campestris* pv. *campestris* reveals a GntR family transcriptional regulator controlling hypersensitive response and virulence. Mol Plant Microbe Interact 24: 1027–1039. 21615202 10.1094/MPMI-08-10-0180

[33] Slater H, Alvarez-Morales A, Barber CE, Daniels MJ, Dow JM (2000) A two-component system involving an HD-GYP domain protein links cell-cell signalling to pathogenicity gene expression in *Xanthomonas campestris* . Mol Microbiol 38: 986–1003. 11123673 10.1046/j.1365-2958.2000.02196.x

[34] Sambrook J, Fritsch EF, Maniatis T (1989) Molecular cloning: a laboratory manual. 2^nd^ Edition Volumes 1, 2 and 3.

[35] Twomey KB, O'Connell OJ, McCarthy Y, Dow JM, O'Toole GA, Plant BJ, Ryan RP (2012) Bacterial cis-2-unsaturated fatty acids found in the cystic fibrosis airway modulate virulence and persistence of *Pseudomonas aeruginosa* . ISME J 26: 939–950. 10.1038/ismej.2011.167 PMC3329111 22134647

[36] An S-Q, Allan JH, McCarthy Y, Febrer M, Dow JM, Ryan RP (2014) The PAS domain-containing histidine kinase RpfS is a second sensor for the diffusible signal factor of *Xanthomonas campestris* . Mol Microbiol 92: 586–597. 24617591 10.1111/mmi.12577 PMC4159695

[37] McCarthy Y, Yang L, Twomey KB, Sass A, Tolker-Nielsen T, Mahenthiralingam E, Dow JM, Ryan RP (2010) A sensor kinase recognizing the cell-cell signal BDSF (cis-2-dodecenoic acid) regulates virulence in *Burkholderia cenocepacia* . Mol Microbiol 77: 1220–1236. 20624216 10.1111/j.1365-2958.2010.07285.x

[38] Anderson GG, Moreau-Marquis S, Stanton BA, O'Toole GA (2008) *In vitro* analysis of tobramycin-treated *Pseudomonas aeruginosa* biofilms on cystic fibrosis-derived airway epithelial cells. Infect Immun 76: 1423–1433. 18212077 10.1128/IAI.01373-07 PMC2292855

[39] Moreau-Marquis S, Stanton BA, O'Toole GA (2008) *Pseudomonas aeruginosa* biofilm formation in the cystic fibrosis airway. Pulm Pharmacol Therap 21: 595–599. 18234534 10.1016/j.pupt.2007.12.001 PMC2542406

