# Supplementary figures and images for: Novel Cyclic di-GMP Effectors of the YajQ Protein Family Control Bacterial Virulence
Source: PLoS Pathog. 2014 Oct 16;10(10):e1004429. doi: 10.1371/journal.ppat.1004429 (PMC4199771; doi:10.1371/journal.ppat.1004429)

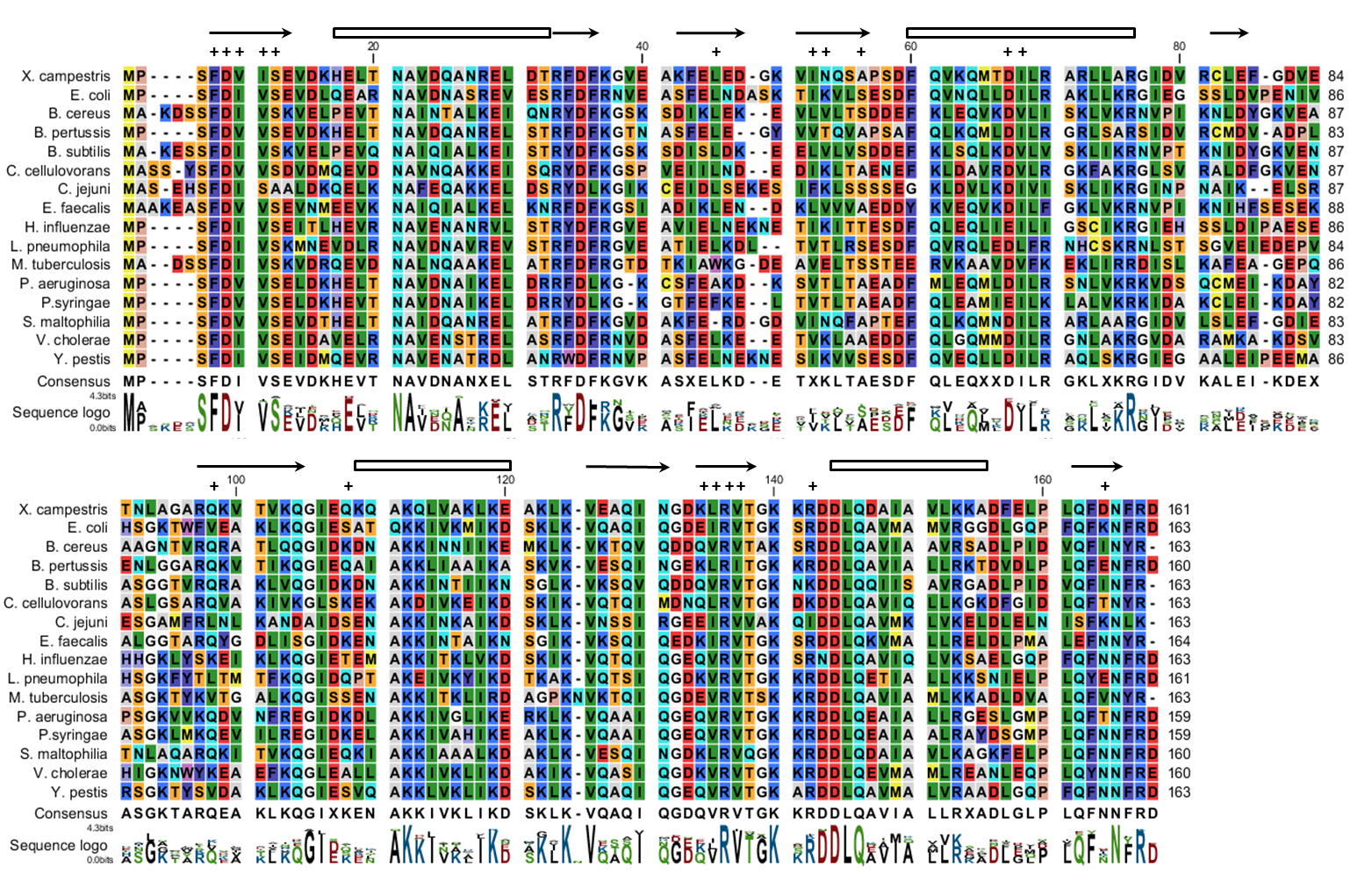

Supplement: Figure S1 — Sequence alignment of proteins from the YajQ family using CLC workbench software. Residues with similar properties are boxed within the same color. Key: Xanthomonas campestris (XC_3703), Escherichia coli (YajQ), Bacillus cereus (BCK_02545), Bordetella pertussis (BP1193_10170), Bacillus subtilis (YitK), Clostridium cellulovorans (Clocel_3875), C. jejuni (BN867_03480), Enterococcus faecalis (EFD32_0973), Haemophilus influenzae (R2846_1298), Legionella pneumophila (LPE509_01999), Mycobacterium tuberculosis (MT0592), Pseudomonas aeruginosa (PA4395), Pseudomonas syringae (PSPPH_4093), Stenotrophomonas maltophilia (Smlt4090), Vibrio cholerae (VC_1508), Yersinia pestis (YPC_3455). Sequence alignment of proteins from the YajQ family. Consensus residues are listed below the alignment. The sequence logo illustrates the conservation between sequences. Where the height of each letter reflects the relative frequency of the corresponding amino acid at that position; the overall height of the column reflects the degree of sequence conservation at that position. On the top of the sequences are indicated the secondary structure elements as Quick2D (http://toolkit.tuebingen.mpg.de/quick2_d) and the residues predicted to be involved in interaction with tRNA are labeled with “+”. (TIF) [file ppat.1004429.s001.tif]

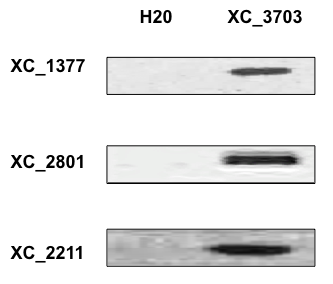

Supplement: Figure S2 — Far-Western analysis showing interactions of XC_3703 with XC_1377, XC_2211 and XC_2801. Lysates of Xcc overexpressing the XC_3703 protein were separated by SDS polyacrylamide gel electrophoresis, transferred to nitrocellulose membranes and separate blots were probed with His6 tagged XC_1377, XC_2211 or XC_2801 which was then detected with an anti-His6 antiserum. The left lane on each blot (H2O) represents a blank control with no His-tagged protein. Far–western signals were seen with XC_1377, XC_2801 and XC_2211. (TIF) [file ppat.1004429.s002.tif]

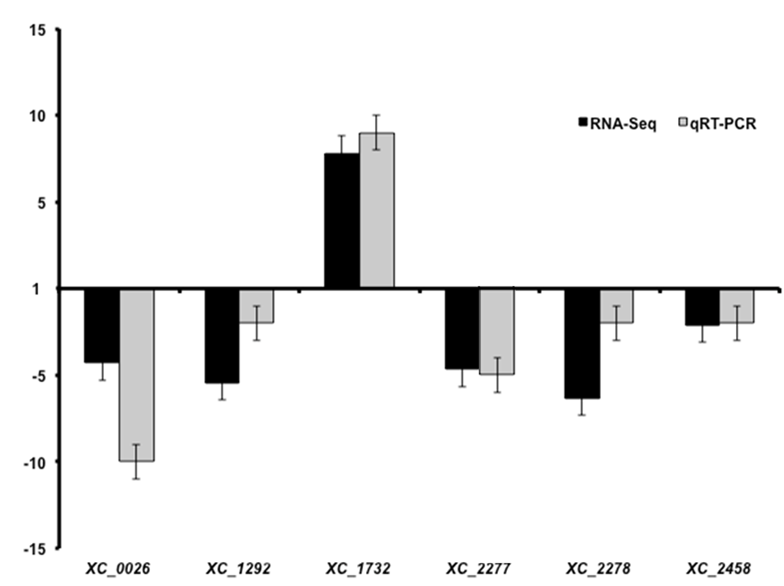

Supplement: Figure S3 — Differential expression of selected genes implicated in virulence or biofilm formation in the XC_2801 deletion mutant and wild-type as determined by RNA-Seq (dark grey) and qRT–PCR (light grey). Mutation of XC_2801 affected transcript levels of XC_0026 (cellulase), XC_1292 (endoproteinase), XC_1732 (glycosyltransferase), XC_2277 (flagellar biosynthesis protein), XC_2278 (flagellar biosynthesis protein), XC_2458 (endomannosidase). The qRT–PCR data were normalised to 16S rRNA and is presented as the fold change with respect to the wild-type for each gene. Data (means ± standard deviation) are derived from four independent biological experiments. The complete RNA-Seq data set is detailed in Table S3; S4. (TIF) [file ppat.1004429.s003.tif]

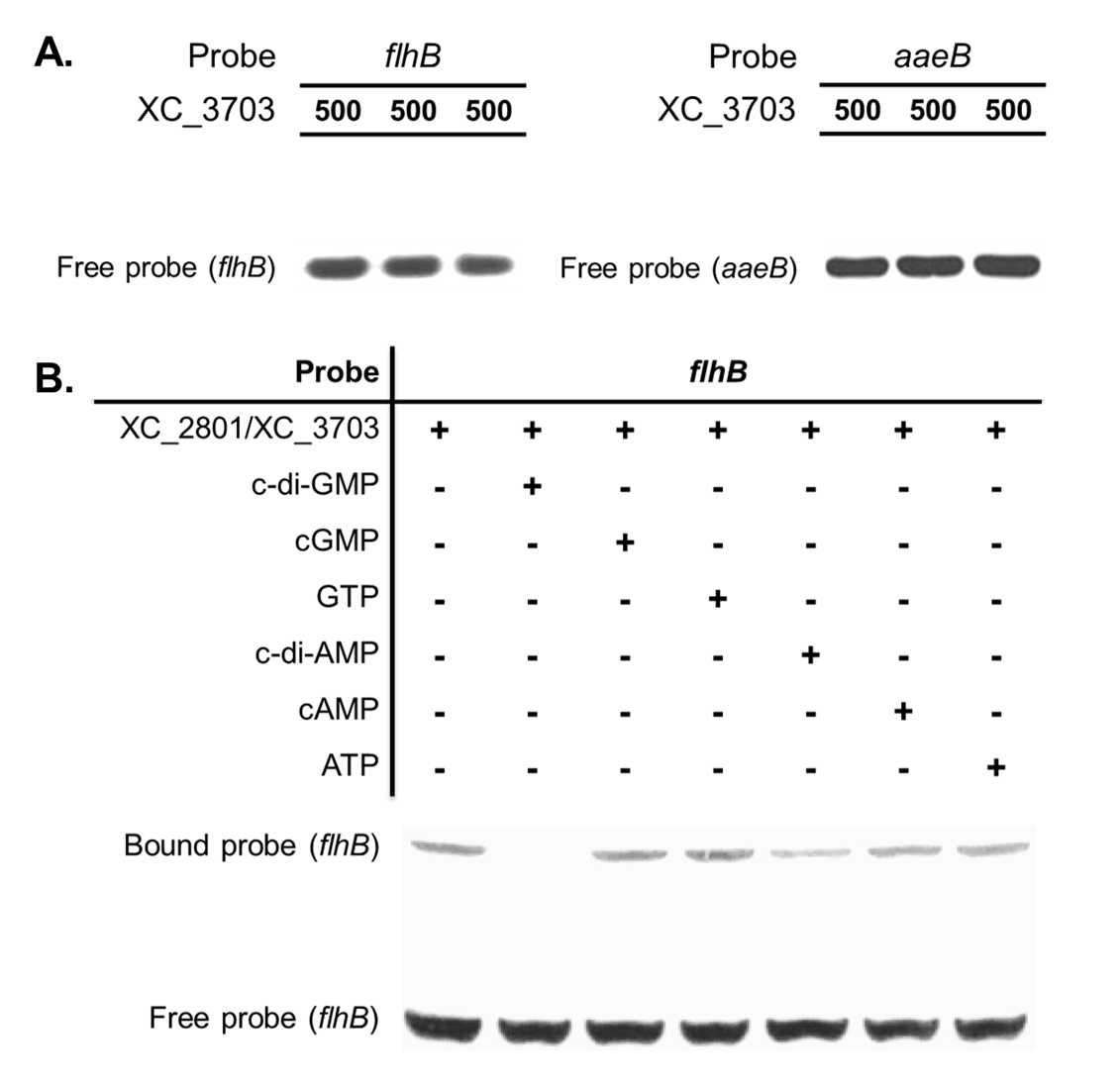

Supplement: Figure S4 — (A) Promoter regions of flhB and aaeB showed no mobility in presence of XC_3703 alone as assessed by electromobility shift assay (EMSA). Each lane contained 1.5 nM DIG-labelled Probe DNA, and in addition to 500 nM of purified His6-tag XC_3703 protein. (B) Binding of XC_2801//XC_3703 complex to the flhBA promoter in the presence of various nucleotides. EMSA assessment of the impact of the presence of nucleotides AMP, GTP, cyclic GMP, cyclic di-AMP, ATP or cyclic AMP at 10 µM on the binding of XC_2801//XC_3703 to the flhBA promoter. DIG-labelled promoter fragments were incubated with purified XC_2801//XC_3703 proteins in the absence or presence of nucleotide as indicated. (TIF) [file ppat.1004429.s004.tif]

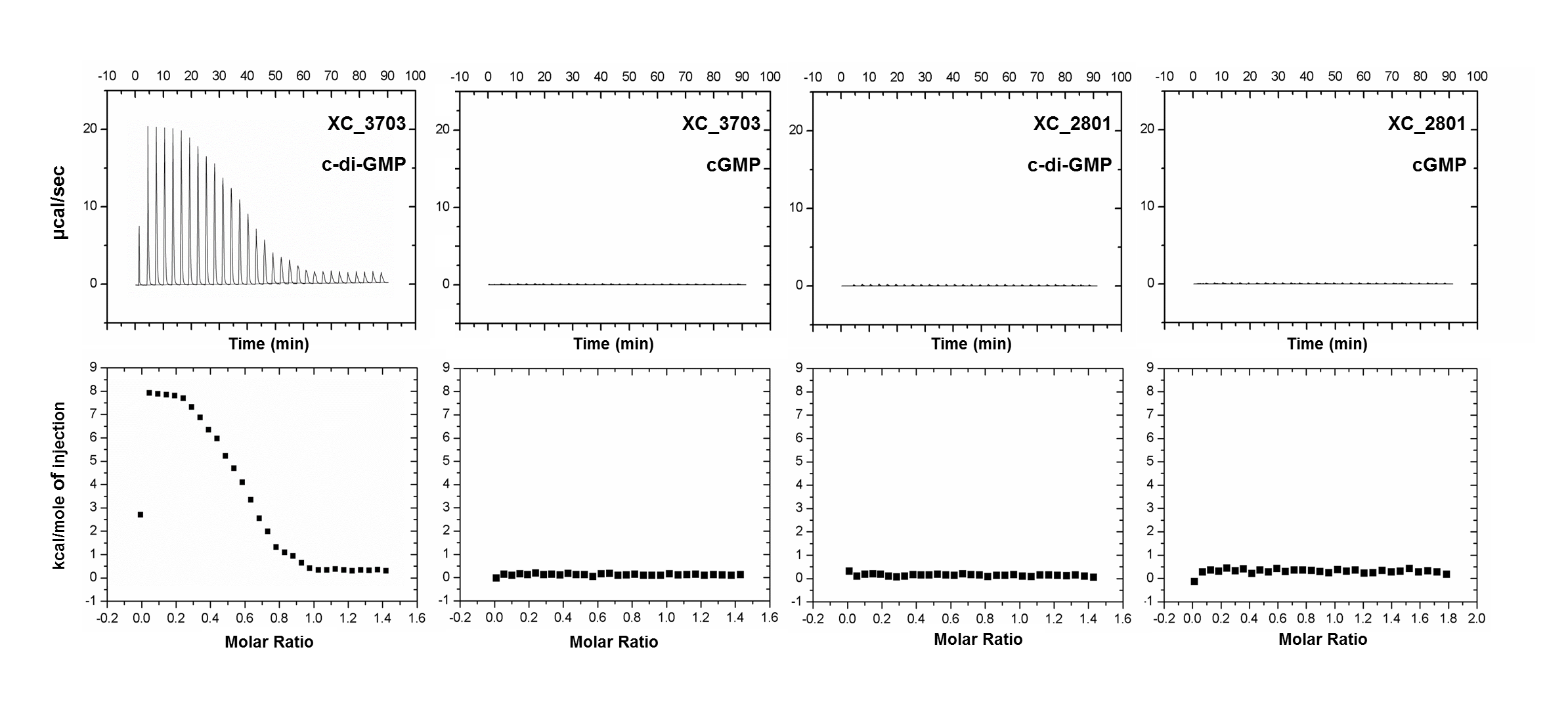

Supplement: Figure S5 — Assessment of binding of cyclic di-GMP and cyclic GMP by XC_2801 using isothermal titration calorimetry. Panels show the integrated data obtained from the raw data, after subtracting the heat of dilution. Experimental data were fitted using the MicroCal ORIGIN version 7.0 software. XC_2801 has no apparent affinity for cyclic di-GMP. (TIF) [file ppat.1004429.s005.tif]

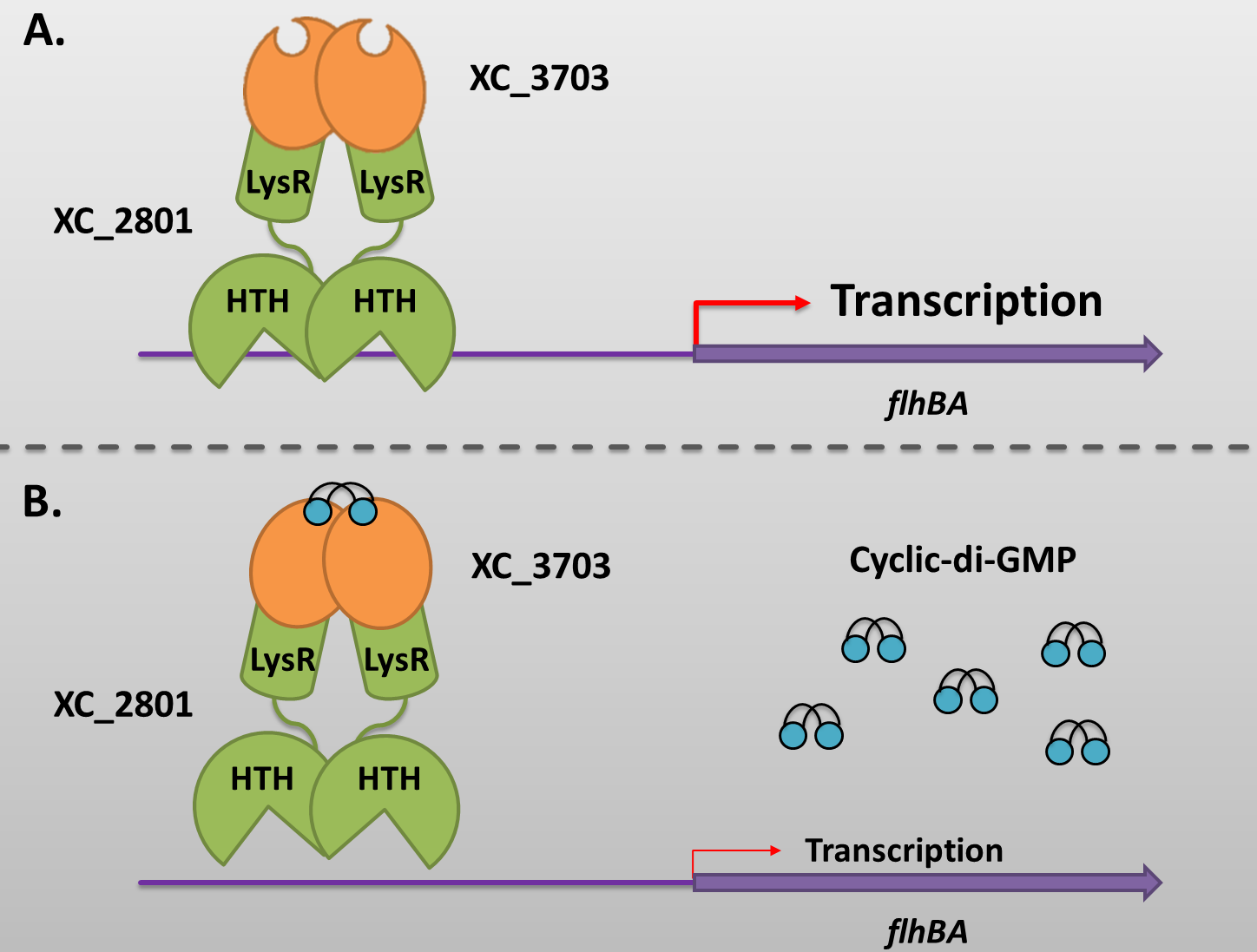

Supplement: Figure S6 — Model for the regulation of gene expression by XC_2801, XC_3703 and cyclic di-GMP. DNA binding and the transcriptional activity of XC_2801 is promoted by binding of XC_3703 to the LysR_substrate-binding domain. The binding of cyclic di-GMP to XC_3703 inhibits this protein-protein complex from interaction with DNA, thereby leading to a reduction in gene transcription. We cannot exclude however that the binding of cyclic di-GMP by XC_3703 prevents the interaction with XC_2801, which cannot bind the promoter alone. XC_3703 alone does not bind to the promoter DNA and XC_2801 does not bind cyclic di-GMP. (TIF) [file ppat.1004429.s006.tif]
