# Supplementary material for: Novel Cyclic di-GMP Effectors of the YajQ Protein Family Control Bacterial Virulence
Source: PLoS Pathog. 2014 Oct 16;10(10):e1004429. doi: 10.1371/journal.ppat.1004429 (PMC4199771; doi:10.1371/journal.ppat.1004429)
Supplement: Table S1 — Proteins that were identified in three cyclic di-GMP pull down experiments. (DOCX) [file ppat.1004429.s007.docx]

**Table S1.** Proteins that were identified in three cyclic di‑GMP pull down experiments.

| **XC number^a^** | **Gene designation** | **MOWSE score** ^b^ | **Description** ^c^ | **Comment** |
| --- | --- | --- | --- | --- |
| XC_0486 | *clp* | 85 | CRP/FNR family transcriptional regulator | Experimentally demonstrated to bind cyclic di-GMP |
| XC_0965 |  | 71 | PilZ domain containing protein | Experimentally demonstrated to bind cyclic di-GMP |
| XC_1036 |  | 62 | GGDEF domain containing protein | Experimentally demonstrated diguanylate cyclase; predicted I-site for cyclic di-GMP-binding |
| XC_2641 |  | 102 | Hypothetical protein |  |
| XC_3221 |  | 53 | A type IV fimbriae assembly protein containing a PilZ domain | Experimentally demonstrated to bind cyclic di-GMP |
| XC_3703 | *yajQ* | 93 | Nucleotide-binding protein of the YajQ family |  |
| XC_4309 |  | 34 | Amino acid/polyamine antiporter transporter |  |

^a^ Proteins are listed according to ascending XC numbers.

^b^ Probability of the protein identify formed the basis of MASCOT (Search algorithm for Mass Spectral proteomics peaklists).

^c^ Domains highlighted as predicted by Pfam.
