# Supplementary material for: Novel Cyclic di-GMP Effectors of the YajQ Protein Family Control Bacterial Virulence
Source: PLoS Pathog. 2014 Oct 16;10(10):e1004429. doi: 10.1371/journal.ppat.1004429 (PMC4199771; doi:10.1371/journal.ppat.1004429)
Supplement: Table S2 — Binding constants of XC_3703 and other YajQ-like proteins for different nucleotides as determined by isothermal titration calorimetry. (DOCX) [file ppat.1004429.s008.docx]

**Table S2.** Binding constants of XC_3703 and other YajQ-like proteins for different nucleotides as determined by isothermal titration calorimetry.

| **Protein target** | **Ligand** | **Kd (μM)** |
| --- | --- | --- |
|  |  |  |
| XC_3703 | ATP | 3.00 (± 0.05) x 10^3^ |
|  | GTP | 1.30 (± 0.05) x 10^4^ |
|  | Cyclic-di-GMP | 2.20 (± 0.51) |
|  | Cyclic-di-AMP | 0.90 (± 0.07) x 10^3^ |
|  | Cyclic GMP | N.D. |
|  | Cyclic AMP | N.D. |
|  |  |  |
| YajQ | ATP | 28.23 (± 4.44) |
|  | GTP | 34.32 (± 3.12) |
|  | Cyclic-di-GMP | N.D. |
|  | Cyclic-di-AMP | N.D. |
|  | Cyclic GMP | N.D. |
|  | Cyclic AMP | N.D. |
|  |  |  |
| PA4395 | ATP | 11.41 (± 0.08) x 10^4^ |
|  | GTP | 12.11 (± 0.03) x 10^4^ |
|  | Cyclic-di-GMP | 6.98 (± 1.13) |
|  | Cyclic-di-AMP | N.D. |
|  | Cyclic GMP | N.D. |
|  | Cyclic AMP | N.D. |
|  |  |  |
| Smlt_4090 | ATP | N.D. |
|  | GTP | N.D. |
|  | Cyclic-di-GMP | 7.91 (± 2.11) |
|  | Cyclic-di-AMP | N.D. |
|  |  |  |
|  |  |  |
|  |  |  |
| BCK_02545 | ATP | 40.82 (± 7.91) |
|  | GTP | 37.53 (± 5.01) |
|  | Cyclic-di-GMP | 1.15 (± 0.01) x 10^4^ |
|  | Cyclic-di-AMP | 3.12 (± 0.02) x10^4^ |
|  | Cyclic GMP | N.D. |
|  | Cyclic AMP | N.D. |
|  |  |  |
| Clocel_3875 | ATP | 21.12 (± 4.13) |
|  | GTP | 30.96 (± 2.91) |
|  | Cyclic-di-GMP | N.D. |
|  | Cyclic-di-AMP | N.D. |
|  | Cyclic GMP | N.D. |
|  | Cyclic AMP | N.D. |

Kd is the dissociation constant.

N.D. Not detected.
