# Supplementary material for: Novel Cyclic di-GMP Effectors of the YajQ Protein Family Control Bacterial Virulence
Source: PLoS Pathog. 2014 Oct 16;10(10):e1004429. doi: 10.1371/journal.ppat.1004429 (PMC4199771; doi:10.1371/journal.ppat.1004429)
Supplement: Table S3 — Summary of sequencing data for the Xcc cDNA samples analysed. (DOCX) [file ppat.1004429.s009.docx]

**Table S3.** Summary of sequencing data for the *Xcc* cDNA samples analyzed.

| **Sequenced sample*** | **Average read size, bp** | **No. of total mapped reads** | **No. of total mapped bps (× 106)** | **No. of genes mapped** | **rRNA reads (% of all mapped reads)** | **otherRNA reads (% of all mapped reads)** |
| --- | --- | --- | --- | --- | --- | --- |
| Wild-type (8004) [1] | 42 | 63,819,059 | 52,568,676 | 4,318 | 0.45 | 1.48 |
| Wild-type (8004) [2] | 43 | 55,983,696 | 73,975,229 | 4,316 | 0.31 | 10.4 |
| Wild-type (8004) [3] | 49 | 59,888,533 | 79,860,858 | 4,319 | 0.29 | 7.3 |
| XC_3703[1] | 31 | 28,061,243 | 68,251,833 | 4,318 | 0.33 | 2.34 |
| XC_3703[2] | 46 | 167,951,087 | 105,137,352 | 4,319 | 0.45 | 0.97 |
| XC_3703[3] | 41 | 83,975,543 | 56,984,837 | 4,317 | 0.26 | 3.11 |
| XC_2801 [1] | 51 | 46,199,740 | 35,045,784 | 4,314 | 0.14 | 0.87 |
| XC_2801 [2] | 55 | 64,732,348 | 44,578,617 | 4,318 | 0.3 | 1.45 |
| XC_2801 [3] | 39 | 36,363,450 | 34,403,186 | 4,318 | 0.11 | 11.1 |
|  |  |  |  |  |  |  |
| **Average total** | **43** | **67,441,633** | **61,200,708** | **4,317** | **0.29** | **4.51** |

*Indicates the *Xanthomonas campestris* strain and biological replicate number [1 or 2 or 3].
