# Supplementary material for: Novel Cyclic di-GMP Effectors of the YajQ Protein Family Control Bacterial Virulence
Source: PLoS Pathog. 2014 Oct 16;10(10):e1004429. doi: 10.1371/journal.ppat.1004429 (PMC4199771; doi:10.1371/journal.ppat.1004429)
Supplement: Table S4 — List of genes expressed in XC_2801 and XC_3703 mutant backgrounds compared to wild-type. Significantly differentially expressed genes (fold change ≥3) were determined using Cufflinks after Benjamini-Hochberg correction. The fold change is the ratio of mutant fragments per kilobase of exon per million fragments mapped (FPKM) to wild-type FPKM. (DOCX) [file ppat.1004429.s010.docx]

**Table S4.** List of genes differentially expressed in *XC_2801* and *XC_3703* mutant backgrounds compared to wild-type. Significantly differentially expressed genes (fold change ≥ 3) were determined using Cufflinks after Benjamini-Hochberg correction. The fold change is the ratio of mutant fragments per kilobase of exon per million fragments mapped (FPKM) to wild-type FPKM.

| **ID** | **Definition** | ***XC_2801*** | ***XC_3703*** |
| --- | --- | --- | --- |
| XC_0026 | cellulase | -4.30 | -4.64 |
| XC_0027 | cellulase |  | -7.20 |
| XC_0063 | regulatory protein cII |  | -4.37 |
| XC_0128 | hypothetical protein |  | -7.75 |
| XC_0129 | hypothetical protein |  | -4.55 |
| XC_0130 | hypothetical protein |  | -5.30 |
| XC_0131 | hypothetical protein |  | -5.18 |
| XC_0132 | deoxycytidylate deaminase |  | -9.69 |
| XC_0249 | hypothetical protein |  | 3.35 |
| XC_0250 | hypothetical protein |  | 3.47 |
| XC_0347 | hypothetical protein |  | -4.87 |
| XC_0723 | hypothetical protein |  | -3.13 |
| XC_0727 | hypothetical protein |  | -3.20 |
| XC_0817 | hypothetical protein |  | -5.90 |
| XC_0860 | hypothetical protein |  | -3.92 |
| XC_0861 | hypothetical protein |  | -4.16 |
| XC_0863 | hypothetical protein |  | -3.79 |
| XC_0867 | hypothetical protein |  | -5.04 |
| XC_0868 | VirB6 protein |  | -4.10 |
| XC_0916 | BlaI family transcriptional regulator |  | -4.21 |
| XC_1019 | hypothetical protein |  | -11.12 |
| XC_1020 | hypothetical protein |  | -8.44 |
| XC_1022 | regulatory protein bphR |  | -3.89 |
| XC_1028 | hypothetical protein |  | -5.61 |
| XC_1029 | hypothetical protein |  | -4.75 |
| XC_1030 | hypothetical protein |  | -5.22 |
| XC_1058 | pilin |  | -7.04 |
| XC_1059 | pilin |  | -4.72 |
| XC_1165 | TonB-dependent receptor |  | -3.49 |
| XC_1166 | glucokinase |  | -3.11 |
| XC_1292 | endoproteinase ArgC | -5.45 | -3.95 |
| XC_1298 | pectate lyase II |  | -4.12 |
| XC_1300 | quinol oxidase subunit I |  | 14.72 |
| XC_1301 | quinol oxidase subunit II |  | 8.62 |
| XC_1332 | DNA transport competence protein |  | -3.59 |
| XC_1391 | hypothetical protein | -6.31 | -4.82 |
| XC_1515 | extracellular protease |  | -8.31 |
| XC_1682 | outer membrane lipoprotein |  | -3.43 |
| XC_1711 | hypothetical protein |  | 4.04 |
| XC_1732 | hypothetical protein | 7.82 | 12.23 |
| XC_2013 | sensor kinase |  | -5.40 |
| XC_2055 | hypothetical protein |  | -3.86 |
| XC_2087 | tannase |  | -16.82 |
| XC_2088 | hypothetical protein |  | -19.03 |
| XC_2123 | hypothetical protein |  | -3.52 |
| XC_2232 | flgA,flagellar basal body P-ring biosynthesis protein FlgA | -3.73 |  |
| XC_2234 | flgB, flagellar basal-body rod protein FlgB | -9.65 | -7.03 |
| XC_2239 | flgG, flagellar basal body rod protein FlgG | -7.49 | -6.11 |
| XC_2240 | flgH, flagellar basal body L-ring protein | -6.54 | -6.07 |
| XC_2241 | flgI, flagellar basal body P-ring biosynthesis protein FlgA | -4.37 | -4.23 |
| XC_2242 | flgJ, flagellar rod assembly protein/muramidase FlgJ | -3.59 |  |
| XC_2243 | flgK, flagellar hook-associated protein FlgK | -4.05 | -3.76 |
| XC_2244 | flgL, flagellar hook-associated protein FlgL | -3.53 | -3.33 |
| XC_2251 | RNA polymerase sigma-54 factor | -4.32 |  |
| XC_2259 | flagellar protein | -4.59 | -5.59 |
| XC_2260 | fliF, lar MS-ring protein | -4.12 | -3.71 |
| XC_2261 | flagellar protein | -4.43 | -3.89 |
| XC_2264 | flagellar FliJ protein | -3.18 | -3.45 |
| XC_2267 | fliM, flagellar motor switch protein FliM |  | -5.61 |
| XC_2269 | flagellar protein |  | -3.82 |
| XC_2272 | flagellar biosynthesis | -3.58 | -4.48 |
| XC_2277 | flhB, flagellar biosynthesis protein FlhB | -3.19 | -3.39 |
| XC_2278 | flhA, flagellar biosynthesis protein FlhA | -3.45 | -3.51 |
| XC_2279 | flagellar biosynthesis regulator FlhF | -8.42 | -5.38 |
| XC_2294 | arsenite efflux pump ACR3 |  | -3.96 |
| XC_2295 | high-affinity Fe2+/Pb2+ permease |  | -6.23 |
| XC_2403 | MchC protein |  | -3.34 |
| XC_2405 | transport transmembrane protein |  | -3.99 |
| XC_2406 | hypothetical protein |  | -4.76 |
| XC_2407 | hypothetical protein |  | -4.13 |
| XC_2408 | hydroxyproline-rich glycoprotein DZ-HRGP |  | -3.52 |
| XC_2409 | hypothetical protein |  | -3.72 |
| XC_2410 | hypothetical protein |  | -5.15 |
| XC_2413 | NTPase VagA |  | -3.91 |
| XC_2418 | hypothetical protein | -3.75 | -122.77 |
| XC_2419 | hypothetical protein |  | -54.63 |
| XC_2420 | hypothetical protein |  | -28.66 |
| XC_2421 | phage-related integrase |  | -3.75 |
| XC_2422 | hypothetical protein |  | -5.83 |
| XC_2423 | hypothetical protein |  | -5.24 |
| XC_2424 | hypothetical protein |  | -19.83 |
| XC_2426 | RNA-directed DNA polymerase |  | -3.87 |
| XC_2427 | hypothetical protein |  | -4.22 |
| XC_2428 | ankyrin repeat-containing protein |  | -3.55 |
| XC_2429 | hypothetical protein |  | -3.29 |
| XC_2437 | hypothetical protein |  | -14.25 |
| XC_2438 | plasmid-like protein |  | -25.14 |
| XC_2444 | hypothetical protein |  | -3.24 |
| XC_2456 | two-component system sensor protein |  | 7.50 |
| XC_2457 | two-component system regulatory protein |  | 3.89 |
| XC_2458 | mannan endo-1,4-beta-mannosidase | -5.71 | -5.24 |
| XC_2474 | hypothetical protein |  | -11.97 |
| XC_2475 | sensor kinase |  | -4.80 |
| XC_2608 | hypothetical protein |  | -3.23 |
| XC_2609 | hypothetical protein |  | -3.60 |
| XC_2610 | hypothetical protein |  | -4.98 |
| XC_2620 | hypothetical protein |  | -3.82 |
| XC_2632 | hypothetical protein | 3.38 |  |
| XC_2633 | hypothetical protein | 3.68 |  |
| XC_2634 | hypothetical protein | 4.04 |  |
| XC_2638 | phage-related integrase |  | -4.11 |
| XC_2723 | transcriptional regulator |  | 3.48 |
| XC_2724 | 5-methyltetrahydrofolate--homocysteine methyltransferase |  | 3.07 |
| XC_2848 | asnB, asparagine synthetase B | -5.60 | -8.64 |
| XC_2857 | protein U | -8.61 | -5.18 |
| XC_2858 | pili assembly chaperone | -4.69 |  |
| XC_2859 | outer membrane usher protein FasD | -3.14 |  |
| XC_2860 | hypothetical protein | -3.35 |  |
| XC_2898 | hypothetical protein |  | -3.12 |
| XC_2938 | hypothetical protein |  | -4.49 |
| XC_2977 | 3-hydroxyisobutyrate dehydrogenase | -3.26 |  |
| XC_2978 | enoyl-CoA hydratase |  | -3.02 |
| XC_2979 | enoyl-CoA hydratase | -3.89 | -4.43 |
| XC_2980 | acyl-CoA dehydrogenase | -4.28 | -5.11 |
| XC_2981 | methylmalonate-semialdehyde dehydrogenase | -4.97 |  |
| XC_3035 | DNA mismatch repair protein MutS |  | 3.27 |
| XC_3280 | peptidyl-Asp metalloendopeptidase |  | -3.92 |
| XC_3487 | alpha-amylase | -5.31 | -7.64 |
| XC_3553 | hypothetical protein | 9.97 | 8.72 |
| XC_3554 | hypothetical protein | 6.99 | 10.84 |
| XC_3555 | hypothetical protein | 6.90 | 5.45 |
| XC_3556 | hypothetical protein |  | 11.09 |
| XC_3591 | pectate lyase | -5.08 | -9.47 |
| XC_3645 | hypothetical protein |  | 4.17 |
| XC_3696 | hypothetical protein |  | -3.00 |
| XC_3800 | response regulator |  | -3.75 |
| XC_3805 | hypothetical protein |  | -3.26 |
| XC_3870 | hypothetical protein |  | -3.27 |
| XC_3902 | cytochrome C oxidase assembly protein | -3.04 |  |
| XC_3922 | hypothetical protein |  | -3.60 |
| XC_3951 | glucosyltransferase |  | -3.39 |
| XC_3952 | ATP-dependent serine activating enzyme |  | -3.95 |
| XC_4012 | hypothetical protein |  | -3.29 |
| XC_4043 | hypothetical protein |  | -4.37 |
| XC_4152 | cytochrome C biogenesis protein |  | -15.19 |
| XC_4153 | hypothetical protein | -15.77 | -21.17 |
| XC_4291 | microcystin dependent protein | -3.11 |  |

a: Annotation according to Qian *et al*, (2005).

b: Predicated function based on best BLAST hits searching the bacterial genome database.

c: Fold change in gene expression in selected mutant compared to wild-type strain - log2 scaled fold change of gene expression.
