# Supplementary material for: Novel Cyclic di-GMP Effectors of the YajQ Protein Family Control Bacterial Virulence
Source: PLoS Pathog. 2014 Oct 16;10(10):e1004429. doi: 10.1371/journal.ppat.1004429 (PMC4199771; doi:10.1371/journal.ppat.1004429)
Supplement: Table S5 — Summary of protein–protein interactions involving XC_3703 observed in this study. (DOCX) [file ppat.1004429.s011.docx]

**Table S5.** Summary of protein–protein interactions involving XC_3703 proteins observed in this study.

| **Bait name (residues in bait)^a^** | **Bait gene ^a^** | **Total positive preys sequenced (identified)^b^** | **Specific preys ^a, c^** | **Suggested function** |
| --- | --- | --- | --- | --- |
| XC_3703 (1-161 aa) | XC_3703 | 36 (15) | XC_1377 | octaprenyl-diphosphate synthase |
|  |  |  | XC_1554 | ribosomal protein S12 methylthiotransferase |
|  |  |  | XC_1654 | phenylalanyl-tRNA synthetase subunit alpha |
|  |  |  | XC_1655 | phenylalanyl-tRNA synthetase subunit beta |
|  |  |  | XC_2001 | CDP-diacylglycerol-glycerol-3-phosphate 3-phosphatidyltransferase |
|  |  |  | XC_2209 | recombination factor protein, RarA |
|  |  |  | XC_2211 | outer-membrane lipoprotein carrier protein |
|  |  |  | XC_2278 | flagellar biosynthesis protein, FlhA |
|  |  |  | XC_2281 | RNA polymerase sigma factor, FliA |
|  |  |  | XC_2492 | hypothetical protein |
|  |  |  | XC_2736 | GntR family transcriptional regulator |
|  |  |  | XC_2801 | transcriptional regulator |
|  |  |  | XC_3555 | hypothetical protein |
|  |  |  | XC_3612 | glucose-1-phosphate thymidylyltransferase |
|  |  |  | XC_4328 | tRNA modification GTPase, TrmE |

**^a^**The gene designation as defined by *Xcc* genome (GEO accession: GSE5087).

**^b^**The number of positive colonies that grew on selective plates. The number of colonies that yielded usable sequence data is indicated in brackets.

**^c^**Genes identified from sequenced preys using BLAST analysis of the *Xcc* genome (GEO accession: GSE5087).
