# Supplementary material for: Novel Cyclic di-GMP Effectors of the YajQ Protein Family Control Bacterial Virulence
Source: PLoS Pathog. 2014 Oct 16;10(10):e1004429. doi: 10.1371/journal.ppat.1004429 (PMC4199771; doi:10.1371/journal.ppat.1004429)
Supplement: Table S6 — Strains and plasmids used in this study. (DOCX) [file ppat.1004429.s012.docx]

**Table S6.** Strains and plasmids used in this study.

| **Strain or plasmid** | **Relevant characteristics** | **Source or Reference** |
| --- | --- | --- |
| Xcc 8004 | *X. campestris* wild-type Rif^R^ | 31 |
| Xcc 8005/ pPH1JI | Gm^R^ | 31 |
| *XC_3703* | As 8004, but *XC_3703* is deleted*,* Rif^R^ | This study |
| *XC_3703*Km | As 8004, but *XC_3703*::pK18*mob* Rif^R^, Km^R^ | This study |
| *XC_2801* | As 8004, but *XC_2801* is deleted*,* Rif^R^ | This study |
| *XC_2801*Km | As 8004, but *XC_2801*::pK18*mob* Rif^R^, Km^R^ | This study |
| *XC_3703* (c3703) | Complemented *XC_3703* mutant Rif^R^, Km^R^ | This study |
| *XC_3703* (p3703) | *XC_3703* mutant expressing gene encoding his-tagged XC_3703 protein from pLAFR3 | This study |
| *XC_2801* (c2801) | Complemented *XC_2801* mutant Rif^R^, Km^R^ | This study |
| *XC_2801* (p2801) | XC_*2801* mutant expressing gene encoding his-tagged XC_2801 protein from pLAFR3 | This study |
|  |  |  |
| PA14 | *P. aeruginosa,* wild-type, UCBPP-PA14, human isolate | 38 |
| PA4395 | As PA14, but PA4395 is deleted. | This study |
| K279a | *S. maltophilia* wild-type, Clinical isolate | 18 |
| Smlt4090 | As K279a, but Smlt4090 is deleted. | This study |
|  |  |  |
| pRK2073 | Helper plasmid, Sp^R^ | 31 |
| pK18*mobkan* | *lacZa*, Km^R^ | 31 |
| pK18*mobtet* | *lacZa*, Tc^R^ | 31 |
| pK18*mobsacB* | *sacB*, *lacZa*, KmR allelic exchange  vector | 31 |
| pLAFR1::Tn5gusA5 | Broad-host-range IncP2, cosmid, Km^R^, Tc^R^ | 32 |
| pLAFR3 | Broad-host-range IncP2, cosmid, Tc^R^ | 32 |
| pPH1JI | Sp^R^, Gm^R^ | 32 |
