# Supplementary material for: Novel Cyclic di-GMP Effectors of the YajQ Protein Family Control Bacterial Virulence
Source: PLoS Pathog. 2014 Oct 16;10(10):e1004429. doi: 10.1371/journal.ppat.1004429 (PMC4199771; doi:10.1371/journal.ppat.1004429)
Supplement: Table S7 — Primers used in this study. (DOCX) [file ppat.1004429.s013.docx]

**Table S7.** Primers and synthesized fragments of DNA used in this study.

| **Primer** | **Comment** | **Sequence (5’ to 3’)** |
| --- | --- | --- |
| PG2277F  PG2277R | Primers used to construct pG2277 *gusA* fusion | 5’-GGCTGCAGGTGAAAGTTCCG-3’  5’-TGGGGGGCCGTTGACTGGAT-3’ |
| PG3487F  PG3487R | Primers used to construct pG3487  *gusA* fusion | 5’- CATACCGCTGAGCCATTGTG-3’  5’- CGGGTGTGTCTCCTGATCAG-3’ |
| PG2239F  PG2239R | Primers used to construct pG2239 *gusA* fusion | 5’- GTGCGGTGTCCTCGCCGCTC-3’  5’- TGACCCGATTCCTCGTTAGAC-3’ |
| PG2266F  PG2266R | Primers used to construct pG2262 *gusA* fusion | 5’-GCGCGACATGCTGGGCCAGC-3’  5’- CTTTCTCTTCGGTCTTTTCG-3’ |
| flhB probe | Primers used to generate EMSA probe – *flhB* – 160 bp | 5’-CTTTACCTATTCCCGCCCCC-3’  5’-CCGTCTTCGGACTCGGACAT-3’ |
| aaeB probe | Primers used to generate EMSA probe – *aaeB* – 200 bp | 5’- GGCATCGAACCGGCCGCACG-3’  5’-CGGGTGTGTCTCCTGATCAG-3’ |
| flgG probe | Primers used to generate EMSA probe – *flgG* – 180 bp | 5’- CCTGATCTGCCGCGCAGATTT-3’  5’- CCGGGGATGTGTTCCGGGCA-3’ |
| fliL probe | Primers used to generate EMSA probe – *fliL* – 190 bp | 5’- TTCCCACGGGCCCATGGGCC-3’  5’- GGTGTGCTCCAGGGGAATGC-3’ |
| flhB truncation | Primers used to generate EMSA probe – *flhB* truncation – 155 bp | 5’-CCGAATCCCGATTCCCCATTC-3’  5’-CCTTGCTCGCGGGCTTCGCGC-3’ |
|  |  |  |
| **Construct** | **Comment** | **DNA fragment synthesized** |
| pXC3703 | XC_3703 cloned into pET47b | TTCCCAGGATCCGCCGTCCTTCGACGTGATTTCCGAAGTCGACAAGCACGAACTGACCAATGCGGTGGATCAGGCCAACCGCGAGCTGGACACCCGCTTCGACTTCAAGGGCGTGGAAGCCAAGTTCGAACTGGAAGACGGCAAGGTGATCAACCAGTCCGCGCCCAGCGATTTCCAGGTCAAGCAGATGACCGACATCCTGCGCGCGCGCCTGCTGGCCCGTGGCATCGATGTGCGCGCCCTGGAGTTTGGCGATGTGGAGACCAACCTGGCCGGTGCGCGGCAGAAGGTCACCGTCAAGCAGGGCATCGAGCAGAAGCAGGCCAAGCAGCTGGTGGCCAAGTTGAAGGAAGCCAAGCTCAAGGTCGAAGCGCAGATCAACGGCGACAAGCTGCGCGTCACCGGCAAGAAGCGCGACGACCTGCAGGACGCGATCGCAGTATTGAAGAAGGCCGACTTCGAGCTGCCGCTGCAGTTCGACAATTTCCGCGATTGAAAGCTTACTAT |
| pXC2801 | XC_2801 cloned into pET47b | TTCCCAGGATCCGACCCACGATCTCAACGACACCCTGATCTTCGTCAAGGTGGTCGAACAAGGCAGTTTCATCGCTGCCGCCAACTCGCTCGGCCTGCCCAAGACCACGGTCAGCCGCAAGGTGCAGGAACTGGAAACCCGGCTGGGTGCCCGCCTGCTGCACCGGACCACGCGTCGCATCGGCCTGACCGAAGCCGGCGCGGTCTATCACGAACACTGCCAGCGCATTGCCCGTGAACTGGAAGAAGCCGAAAGCGCCGTCGGCCAATTGCAGTCGGGCCCGCGCGGCTGGTTGCGCTTCACCGTGCCGTACTCGCTGGGCATCACCTGGATCGCGCCGTTGCTGGGCGAATTCCACGCGCAATACCCGGAAATCCAGCTGGACATGCACCTGGGCAACGAGAAGCTGGACTTGATCGGCGGCGAAGCCGATCTGGCCCTGCGCGTGGGTGCCCTGCCCGATTCCAACCTGGTTGCGCGCAAGCTGGGCAGCCTGCGCACGCAGGTGTTTGCCAGCCCGTCTTACATCGAACGCTATGGCGAACCGCTGCATCCGGACGAGTTGCAGTTCCATCGCACGCTCGCCCTGCGCAAGAACCGCAATGTGCACAACAACCGCTTCTTCTGGTCGCTCAGCGACGGCAGCGATGTGCGCGAATTCCCGGTCAATCCGTTGATGGTGGCCAACGACCCGGCCGCGCTCAATGGTGCGGTGCTGTGCGGCGAAGGCTTGTTGCTGACCGGCGATGTGATGGCCAAGCCGTTCGTGCAATCGGGCATGGTGCGGCGTGTACTGGCAGGTTGGACCGGGCCGGAAGTGGACTTCAACGCGGTCTTCGCCGGTGGCCGGTTGGTGTCGCCGAAGGTGCGGGCGTTCGTGGATTTCCTGGTGACACGCCTGAACTTCGACGCCGATTACATGATGGCGCAGTGCCCTGCACGGCTGGCCGCACAGCGCGCCAATGGGGATGCGGAGGTGGAGGTTGAAGTGGAGGCCGAGTTGCGCGCCGAGGGTAAGCGCATTTTGGAAAAAGCTACGGCGTGAAAGCTTACTAT |
| pYAJQ | YajQ cloned into pET47b | TTCCCAGGATCCGCCTTCGTTCGACGTGGTGTCCGAACTGGACAAACACGAGTTGACCAACGCCGTGGACAACGCCATCAAGGAACTGGATCGCCGTTTCGACCTGAAAGGCAAAGCCAGTTTCGAAGCCAAGGACAAGTCGGTGACCCTCACCGCCGAAGCCGATTTCATGCTCGAGCAGATGCTCGACATCCTGCGTTCCAACCTGGTCAAGCGCAAGGTCGACAGCCAGGCCATGGAGATCAAGGATGCCTACCCGTCGGGCAAGGTGGTCAAGCAGGACGTCAACTTCCGCGAGGGCATCGACAAGGACCTGGCGAAGAAGATCGTTGGTCTGATCAAGGAGCGCAAGCTCAAGGTCCAGGCCGCCATCCAGGGCGAGCAGGTGCGCGTCACCGGCAAGAAGCGTGACGATCTGCAGGAGGCCATCGCCCTGCTGCGCGGCGAATCCCTCGGCATGCCGTTGCAGTTCACCAACTTCCGCGATTGAAAGCTTACTAT |
| pPA4395 | PA4395 cloned into pET47b | TTCCCAGGATCCGCCTTCGTTCGACGTGGTGTCCGAACTGGACAAACACGAGTTGACCAACGCCGTGGACAACGCCATCAAGGAACTGGATCGCCGTTTCGACCTGAAAGGCAAAGCCAGTTTCGAAGCCAAGGACAAGTCGGTGACCCTCACCGCCGAAGCCGATTTCATGCTCGAGCAGATGCTCGACATCCTGCGTTCCAACCTGGTCAAGCGCAAGGTCGACAGCCAGGCCATGGAGATCAAGGATGCCTACCCGTCGGGCAAGGTGGTCAAGCAGGACGTCAACTTCCGCGAGGGCATCGACAAGGACCTGGCGAAGAAGATCGTTGGTCTGATCAAGGAGCGCAAGCTCAAGGTCCAGGCCGCCATCCAGGGCGAGCAGGTGCGCGTCACCGGCAAGAAGCGTGACGATCTGCAGGAGGCCATCGCCCTGCTGCGCGGCGAATCCCTCGGCATGCCGTTGCAGTTCACCAACTTCCGCGATTGAAAGCTTACTAT |
| pSMLT4090 | Smlt_4090 cloned into pET47b | TTCCCAGGATCCGCCTTCCTTCGACGTCGTGTCCGAAGTCGACACCCACGAGCTGACCAACGCCATCGACCAGGCCAACCGCGAACTGGCCACCCGCTTCGACTTCAAGGGCGTGGACGCAAAGTTCGAGCGCGACGGCGATGTCATCAATCAGTTCGCGCCGACCGAATTCCAGCTCAAGCAGATGAACGACATCCTGCGTGCTCGCCTGGCCGCGCGCGGCATCGACGTGCTCAGCCTGGAGTTCGGCGACATCGAGACCAACCTGGCCCAGGCCCGGCAGAAGATCACCGTCAAGCAGGGCATCGAGCAGAAGATCGCCAAGAAGATCGCGGCGGCCCTGAAGGACGCCAAGCTGAAGGTGGAAAGCCAGATCAACGGCGACAAGCTGCGCGTGCAGGGCAAGAAGCGCGATGACCTGCAGGACGCCATCGCCGTGCTCAAGGCCGGCAAGTTCGAGCTGCCGCTGCAGTTCAACAATTTCCGCGACTGAAAGCTTACTAT |
| pBCK02545 | BCK_02545 cloned into pET47b | GGATCCATGGCAAAAGATAGTTCTTTTGACATCGTTTCGAAAGTAGAATTACCTGAAGTAACAAACGCAATTAACACTGCATTAAAAGAAATCCAAAACCGATATGACTTTAAAGGAAGTAAAAGTGATATTAAACTTGAGAAAGAAGTACTTGTTTTAACTTCTGACGATGAGTTCAAATTAGAGCAAGTAAAAGACGTTCTTATTTCTAAACTCGTAAAACGTAACGTTCCAATTAAGAACTTAGATTACGGAAAAGTTGAAGCGGCGGCTGGTAACACTGTTCGCCAACGCGCAACACTTCAACAAGGTATCGATAAAGATAACGCAAAAAAAATTAACAATATCATTAAAGAAATGAAATTAAAAGTAAAAACACAAGTACAAGATGATCAAGTACGTGTTACGGCGAAAAGCCGTGATGACTTACAAGCAGTTATCGCAGCAGTTCGTAGCGCTGATTTACCAATTGACGTACAATTTATTAACTACCGCCACCACCACCACCACCACTAAAAGCTT |
| pCLOCEL3875 | Clocel_3875 cloned into pET47b | GGATCCATGGCAAGTTCATATTCTTTTGACGTTGTATCTGACGTTGATATGCAAGAAGTAGATAATGCAGTGAATCAAGCAAAAAAGGAAATTTCTCAAAGATATGATTTTAAAGGAAGTCCTGTCGAAATCATCTTAAATGATGAAGATATAAAATTAACTGCAGAAAATGAATTTAAATTAGACGCTGTTCGTGATGTATTAAGAGGAAAATTCGCTAAGAGAGGGCTTTCAGTAAGAGCTCTTGACTTTGGCAAAGTTGAAAATGCATCTTTAGGATCAGCAAGACAAGTTGCTAAAATAGTTAAAGGTCTTTCTAAAGAAAAGGCTAAGGACATAGTTAAAGAGATTAAAGACAGCAAAATCAAAGTTCAAACTCAAATAATGGATAATCAATTAAGAGTTACAGGTAAAGATAAAGACGATCTTCAAGCTGTTATCCAATTATTAAAAGGCAAAGATTTTGGAATTGATCTTCAATTCACCAACTATAGACACCACCACCACCACCACTAAAAGCTT |
